# Protein Crystallography in Vaccine Research and Development

**DOI:** 10.3390/ijms160613106

**Published:** 2015-06-09

**Authors:** Enrico Malito, Andrea Carfi, Matthew J. Bottomley

**Affiliations:** 1 Protein Biochemistry Department, Novartis Vaccines & Diagnostics s.r.l. (a GSK Company), Via Fiorentina 1, 53100 Siena, Italy; E-Mail: enrico.x.malito@gsk.com; 2 Protein Biochemistry Department, GSK Vaccines, Cambridge, MA 02139, USA; E-Mail: andrea.x.carfi@gsk.com

**Keywords:** crystallization, protein engineering, paratope, meningitis, respiratory syncytial virus (RSV), *Staphylococcus aureus*, human immunodeficiency virus (HIV)

## Abstract

The use of protein X-ray crystallography for structure-based design of small-molecule drugs is well-documented and includes several notable success stories. However, it is less well-known that structural biology has emerged as a major tool for the design of novel vaccine antigens. Here, we review the important contributions that protein crystallography has made so far to vaccine research and development. We discuss several examples of the crystallographic characterization of vaccine antigen structures, alone or in complexes with ligands or receptors. We cover the critical role of high-resolution epitope mapping by reviewing structures of complexes between antigens and their cognate neutralizing, or protective, antibody fragments. Most importantly, we provide recent examples where structural insights obtained via protein crystallography have been used to design novel optimized vaccine antigens. This review aims to illustrate the value of protein crystallography in the emerging discipline of structural vaccinology and its impact on the rational design of vaccines.

## 1. Introduction

Vaccination first began in the 18th Century when Edward Jenner protected humans from smallpox by administering material from humans infected with cowpox. In the 19th Century, Pasteur, Koch, Ramon and Mérieux pioneered the development of live-attenuated and killed vaccines, and inactivated toxins, to protect against rabies, cholera, plague and typhoid. Additional major vaccine developments in the 20th Century provided protection against diphtheria, tetanus, pertussis, polio, several types of meningococcus and pneumococcus, haemophilus influenzae B, hepatitis and influenza. Collectively, these vaccines have eliminated most of the life-threatening childhood diseases that previously caused millions of deaths and severe morbidity, thus rendering vaccination one of the most effective medical interventions in history [1,2]. In the 21st Century, vaccination continues to play a highly significant and expanding role in the control and elimination of disease. Nevertheless, many important disease-causing infections are not yet preventable by vaccination, including, respiratory syncytial virus (RSV), human immunodeficiency virus (HIV), groups A and B streptococcus (GAS, GBS), malaria, tuberculosis and ebola. Moreover, certain vulnerable population groups generally tend to be poorly served by vaccination. Therefore, further research and development of novel vaccines is required to address a plethora of currently unmet, globally-significant medical needs [3].

One of the most important advances in vaccine research over the last 10–15 years was the advent of whole-genome sequencing technology. Genomics drove the development of the “reverse vaccinology” approach, which overcame challenges that had not been resolved via conventional vaccinology [4]. Indeed, it was the whole-genome sequencing of *Neisseria meningitidis* serogroup B that enabled the development of reverse vaccinology for the identification of recombinant antigens for a protein-based vaccine against serogroup B meningococcus [5,6]. Since then, it has become routine to obtain the amino acid sequence of all possible proteins that a pathogen might encode in its genome, which greatly potentiates the early stages of vaccine discovery. However, while all antigen sequences can be readily obtained, this information does not necessarily translate into recombinant antigens with ideal attributes for vaccine development, nor do the sequences necessarily provide insights into antigen structures or functions. Therefore, empirical studies are required in order to optimize the recombinant proteins for development and to provide the degree of antigen characterization desirable prior to embarking on clinical studies—these are the stages where protein crystallography can play a crucial role.

Over the last five years, several examples have been presented where antigen structure determination by X-ray crystallography not only provided a highly-detailed level of antigen characterization but, more importantly, also enabled the design of better antigens. Improvements have encompassed structural modifications that stabilize a desirable conformation of the antigen, or that remove undesirable biological properties such as pore-forming toxin function or catalytic activity, or that modify the surface in order to display preferred epitopes. Indeed, the high sequence variability of antigens on a pathogen surface represents a major hurdle to vaccine design in many cases. To fully understand the antigenic manifestation of such sequence variability, we require insights into the structure, dynamics and conformational variability that the antigen may possess. Structural information can therefore help to identify solutions to these various obstacles, thus facilitating vaccine development.

This review aims to provide a concise survey of several recent advances in vaccine research and development that have been driven by insights obtained from protein crystallography. We present several examples, from both bacterial and viral pathogens, which illustrate how high-resolution structural information can be combined with protein engineering to generate antigens that are safe, immunogenic, broadly-protective, stable, and easy to develop. We also conclude with an outlook of how we expect the field to evolve in the near future.

## 2. Protein Crystallography for Antigen Characterization and Epitope Mapping

One of the major contributions of protein crystallography in vaccine research is the structural characterization of antigens either alone or in complexes with the antigen-binding antibody fragments (Fabs) of neutralizing, or “protective”, monoclonal antibodies (mAbs). The following sections provide an overview of some recent advances and highlights in this field.

### 2.1. Antigen Characterization by X-ray Crystallography

#### 2.1.1. NadA—A Surface-Exposed Meningococcal Adhesin and Vaccine Antigen

It is worthwhile to introduce the pathogen *Neisseria meningitidis*, since research towards a broadly-protective serogroup B meningococcal vaccine has provided interesting examples discussed herein. *N. meningitidis* is a human-specific bacterium that causes severe sepsis and meningococcal meningitis, resulting in death or devastating long-term sequelae, and is responsible for about 50% of bacterial meningitis worldwide, an estimated 1.2 million annual cases [7]. The meningococcal serogroups A, B, C, W and Y are the most common, causing most of the disease, predominantly in infants, young children, and adolescents. Due to the very rapid onset and development of disease, mortality rates among infected individuals are as high as 10%, and sequelae are found in 11%–19% of survivors, despite the availability of antibiotic therapies. Glyco-conjugate vaccines protecting against serogroups A, C, W and Y have shown great efficacy [8], yet development of a conjugate vaccine against serogroup B meningococcus was hampered due to similarity of the B polysaccharide to the “self” neuraminic acid present on human fetal tissues [9]. Consequently, serogroup B meningococcus is responsible for up to 90% of cases of meningitis in Europe and 30%–50% of cases in the United States. However, the first recombinant protein-based meningococcal vaccine, *Bexsero*^®^, was approved in Europe in 2013 [10]. *Bexsero*^®^ has subsequently been approved in over 35 countries worldwide, and two meningococcal vaccines, *Trumenba*^®^ [11] and *Bexsero*^®^, have been approved for use in the United States in late 2014 and early 2015.

*Bexsero^®^* is a multi-component vaccine composed of an outer membrane vesicle component plus three main recombinant meningococcal proteins: the *Neisserial* heparin binding antigen (NHBA), the factor H binding protein (fHbp) and the *Neisseria* adhesin A (NadA), as reviewed previously [12]. Here we briefly describe the structural characterization of NadA, which was not straightforward and therefore also serves to illustrate a number of enabling technologies which may be widely relevant to protein crystallographers in this field.

NadA is a surface-exposed protein belonging to the trimeric autotransporter adhesin (TAA) family. All TAAs are obligate homotrimeric proteins and share a common molecular architecture made of a conserved C-terminal integral membrane β-barrel, which anchors the proteins to the outer membrane, and an N-terminal “passenger” domain that is responsible for adhesion [13]. The passenger domain has a modular architecture and is typically made of a central α-helical domain (the stalk) that forms coiled-coil structures and a distinct N-terminal domain (the head) that is mainly responsible for binding to host cellular receptors.

NadA is known to mediate adhesion to and invasion of epithelial cells [14,15] and to induce bactericidal antibodies in immunized humans [16]. Over 40 different NadA protein sequences have been identified and classified into two main groups, containing six variants overall [17]. The *Bexsero*^®^ vaccine contains NadA variant 3, which shares approximately 90% sequence identity with variants 1 and 2, against which it induces cross-protective antibodies. In contrast, the variants 4, 5 and 6 from group II display only 45%–50% sequence identity with variants from group I, and the two groups are not cross-protective.

Structural studies of NadA were initiated in order to understand the molecular basis for these immunological differences. However, sequence analyses of NadA predicted a long and potentially flexible molecule with a high proportion of α-helical elements likely to form coiled-coils [15], the structure determination of which is notoriously difficult [18]. The NadA variant 3 vaccine construct, which includes the predicted head and the entire stalk, was indeed recalcitrant to crystallization, presumably due to its great length, intrinsic flexibility, and relatively low thermal stability. The latter is typically correlated with poor outcomes in crystallization trials, whereas higher melting-points tend to increase the probability of crystallization [19]. Protein engineering approaches were employed to find expression constructs of NadA fragments that might crystallize more easily [20]. First, differential scanning calorimetry (DSC) was used to screen multiple C-terminal truncation mutants of NadA variant 3 designed with progressively shorter stalks. Next, the design of truncated constructs was translated onto other NadA sequence variants (4 and 5), to explore whether slight differences in amino acid composition could further affect crystallization propensity, an approach previously defined as sequence homolog screening [21,22]. The most thermostable construct obtained by these strategies (NadA variant 5, spanning residues 24–220) crystallized readily and reproducibly. Subsequently, crystals optimized by use of additives diffracted at 2.0 Å resolution [20]. Several attempts to solve the structure by molecular replacement, using these initial native data, were unsuccessful. However, the previous observation that other TAAs with Asn residue substitutions at position *d* of the coiled-coil heptad repeat were able to coordinate ions in the buried core of the hydrophobic coiled-coil [23] inspired attempts to soak halides into crystals of NadA for successful experimental phasing by single anomalous dispersion (SAD).

**Figure 1:**
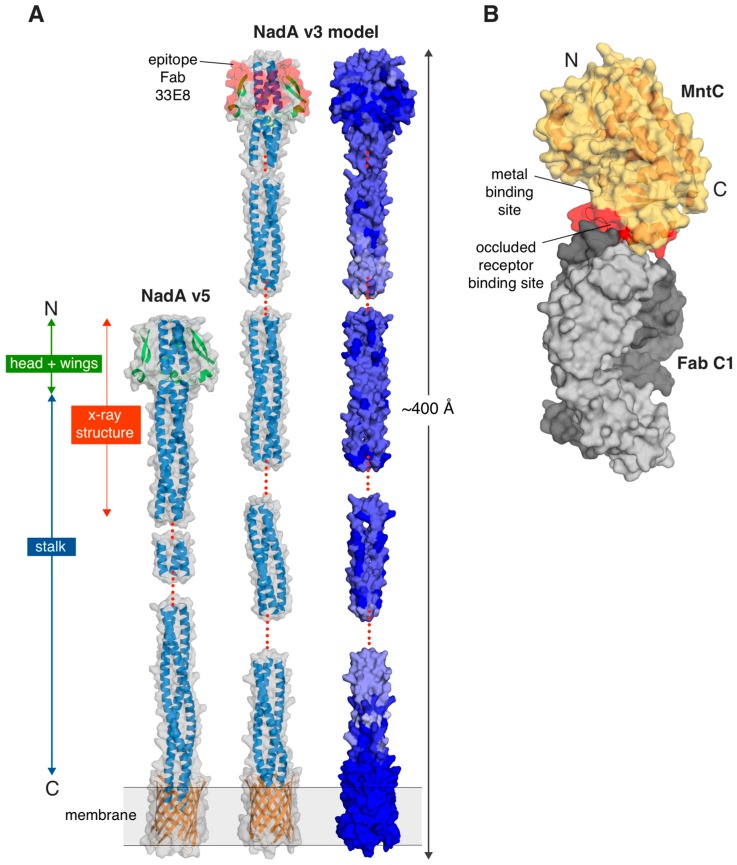
(**A**) The structure of the meningococcal antigen *Neisseria* adhesin A (NadA) variant 5 (pdb 4CJD) is shown on the left, with the region experimentally determined by X-ray crystallography labeled in red. The two main domains of NadA (head + wings and stalk) are labeled with green and blue arrows/boxes, respectively. All other regions were defined by homology modelling, as described previously [20]. A homology model of NadA variant 3 is also shown, with sequence conservation among variants 1–5 depicted as a gradient from light blue (low sequence identity) to dark blue (high sequence identity). The modeled transmembrane anchor is shown in orange. Red dots indicate regions that were not modeled due to lack of predicted coiled-coil periodicity or homology; (**B**) A surface representation of the co-crystal structure of the staphylococcal antigen MntC (semi-transparent light yellow surface and dark yellow cartoon) bound to Fab C1 (light and dark grey surfaces depicting light and heavy chains, respectively) (pdb 4NNP). The binding site of C1 on the surface of MntC (red patch) provides insights into the mechanisms of interaction between MntC and its natural receptor MntB. The Mn^2+^-binding site and the occluded MntB receptor binding sites on MntC are labelled. All figures were prepared using the Pymol software (http://www.pymol.org).

The NadA variant 5 protein exhibits an elongated structure approximately 220 Å-long, and almost exclusively coiled-coil, which runs from the N terminus to the C terminus. The insertion along the coiled-coil of small β-strand structures (residues N49–G84), contribute to make a broader N-terminal region that forms the head domain (Figure 1A) and splits the coiled-coil in two regions. It is remarkable how this sequence interruption apparently does not result in a structural perturbation of the coiled-coil, but forms wing-like structures that protrude from the stalk and pack against the N-terminal coiled-coil helices. Regions of flexibility or disorder were observed along the stalk, with partial electron densities suggesting unwinding of the coiled-coil towards the C terminus, and thus supporting the notion of flexibility as an intrinsic property of this protein.

Elucidation of the structure of NadA variant 5 allowed building of a homology model of the vaccine antigen NadA variant 3 (approximately 50% sequence identity overall), for which high-resolution experimental structural information is not yet available. The homology model was used to visualize sequence and structural differences among the variants, and for epitope mapping of a protective bactericidal mAb (Figure 1A). Interestingly, a surface-plot of the sequence conservation revealed that the largest solvent-exposed patches of highly-conserved NadA residues occur in the head domain, which is also known to be functionally involved in cell adhesion [14,24]. These studies may therefore provide a platform for the design of an optimized “head-only” antigen to be presented in multiple copies on a scaffold. Such an antigen might be able to focus the immune response towards the largest region of potentially cross-reactive epitopes, and ultimately generate a broader immune response.

The structural studies of NadA summarized above represent an example of a rational, biophysically driven approach to determine the 3D structure of a vaccine antigen, which in turn provides useful information to further elucidate both the molecular mechanism of its biological function, and its immunological properties as a vaccine antigen.

#### 2.1.2. Staphylococcal Solute Binding Protein Antigens

Many bacterial pathogens employ solute-binding proteins (SBPs) in nutrient uptake across membranes, as they recognize and deliver substrates to ATP-binding cassette (ABC) importers [25]. In many pathogens, metal ion transport across the membrane is regulated by ABC importers coupled to their SBP partners, and since metals are critical for many biological processes, the inhibition of their acquisition represents an attractive mechanism for developing antibacterial strategies. Similarly, the abundance, variety and surface-exposure of SBPs suggests that they may play key physiological roles and be important virulence factors, thus also becoming targets for vaccine development.

A vaccine discovery project recently identified an SBP protein, FhuD2, as one of five conserved antigens that play important roles in the virulence and pathogenicity of *Staphylococcus aureus* [26]. FhuD2 is a lipoprotein involved in iron uptake and in early stages of invasive *S. aureus* infection [27]. FhuD2 regulates uptake of hydroxamate iron (III) siderophores, which are organic chelators with a very high affinity and specificity for ferric iron. *S. aureus* FhuD2 mediates ligand import through the ABC transporter complex FhuCBG. As part of the characterization and validation of this candidate vaccine antigen, a structural and functional study of FhuD2 was performed, revealing an overall fold similar to known class III SBPs (a bilobate bean-like shape), and iron-loaded ferrichrome bound in a central cleft between the two lobes [28]. Crystallization was initially enabled by stabilization of the protein upon binding to ferrichrome, while in a subsequent study the protein was also crystallized in the apo-form, taking advantage of surface-entropy reducing mutations to promote crystallization [29]. Previously, immunization of mice with FhuD2 was shown to generate protective immunity against diverse clinical isolates of *S. aureus* [27]. To explore whether a more thermostable FhuD2 would induce a more potent antibody response, as might be hypothesized based on the assumption of a better presentation of well-ordered epitopes, mice were immunized with two forms of FhuD2 bound to its stabilizing ligands ferrichrome or coprogen. However, similar degrees of protective immunity were observed when compared to apo-FhuD2, thus validating the unbound form as an effective antigen without the need for additional ligand-mediated stabilization [28].

Another pair of bacterial SBPs that are known immunogens and/or potential vaccine candidates are the manganese transport proteins MntC (*S. aureus*) and its orthologue SitA (*S. pseudintermedius*). MntC is one of two protein antigens in a four-component protein plus polysaccharide vaccine (named SA4Ag) [30] designed to protect broadly against *S. aureus* and currently in early clinical development. As part of the *S. aureus* MntABC importer system, MntC chelates Mn(II) from the host environment and presents it to the integral membrane transporter, MntB. The crystal structures of MntC and SitA were both determined recently and found to be highly similar, as were their metal-binding properties in solution [31,32]. Using a mouse model, active immunizations with MntC were shown to be effective at reducing the bacterial load associated with infections by *S. aureus* and *S. epidermidis*, suggesting that it has the potential to provide protection across multiple staphylococcal species, still to be confirmed in human clinical trials [33]. To learn more about the function of MntC, its crystal structure was determined in complex with an antibody fragment (Fab), obtained from a phage-display library, that blocks Mn^2+^ import (Figure 1B) [34]. Structure-guided mutations of MntC residues in the region recognized by Fab C1 induced hypersensitivity of *S. aureus* to reactive oxygen species, mimicking an mntC null mutant, thus suggesting that the Fab C1 binding site on MntC overlaps with the MntB interaction site. Since a suitable form of the integral membrane-bound MntB protein to use in binding experiments with the MntC proteins was not available, this study showed how the co-crystal structure with a functionally-characterized Fab can be an important tool to indirectly demonstrate the molecular bases of antigen inhibition, in addition to providing important information on the potential development of this antibody as a therapeutic.

In summary, the examples of NadA and MntC provided here illustrate how the availability of antigen structures can aid the interpretation of previous results, guide further investigation, and provide informative starting points for structural vaccinology studies.

### 2.2. Protein Crystallography and High-Resolution B-Cell Epitope Mapping

Almost all current vaccines act via functional antibodies that block infection, bacteremia or viremia [35]. At the first point of host–pathogen interaction, functional antibodies bind to their target epitopes on the pathogen surface proteins (or to epitopes on secreted proteins) and thus initiate the acquired immune defense mechanisms. Epitope mapping is therefore an essential activity in the field of vaccine research, in order to understand the host response to infection or immunization, and has been boosted enormously in recent years by major developments in our abilities to isolate and expand human B cells. The developments enabled the identification of large repertoires of antigen-specific immunoglobulin gene sequences, and thus the easy production of recombinant human antibodies [36,37,38]. Firstly, information about such interactions can improve the chances of being able to select and rationally-design antigen molecules that elicit the desired neutralizing or protective response upon immunization. Secondly, epitope mapping data can indicate which parts of a surface-bound antigen are actually exposed and accessible on the surface of the pathogen, which in turn may provide insights into the functional regions of the protein. Ultimately, an epitope is the full collection of atoms making direct contacts with the antibody, with typical intermolecular distances of up to ~4 Å. Here, we focus on B-cell epitopes, which underlie antigen–antibody interactions and are the crucial molecular determinants of the antibody-mediated immune response.

Many different techniques have been developed to perform epitope mapping, displaying various advantages and limitations, as described previously [39]. For example, although fast and simple, peptide- or fragment-based approaches tend to identify only linear peptide epitopes, thus only partially revealing the immunogenic properties of larger conformational epitopes within their natural three-dimensional contexts. Such approaches have some value, but are unlikely to identify all conformational epitopes recognized by a mAb of interest. In contrast, the crystal structure of an antigen-Fab complex can provide the complete atomic description of an epitope–paratope interface. However, since the success rate of protein crystallization is notoriously unpredictable, efforts to develop additional techniques for conformational epitope mapping have been extensively explored. As a recent development, the more readily-obtained electron microscopy (EM) or hydrogen-deuterium exchange mass spectrometry (HDX-MS) epitope mapping data has been combined with structural data of the antigen, or a structurally-similar homolog, obtained by more lengthy protein crystallographic structural studies, thus forming the basis of a powerful “hybrid method” approach.

**Figure 2:**
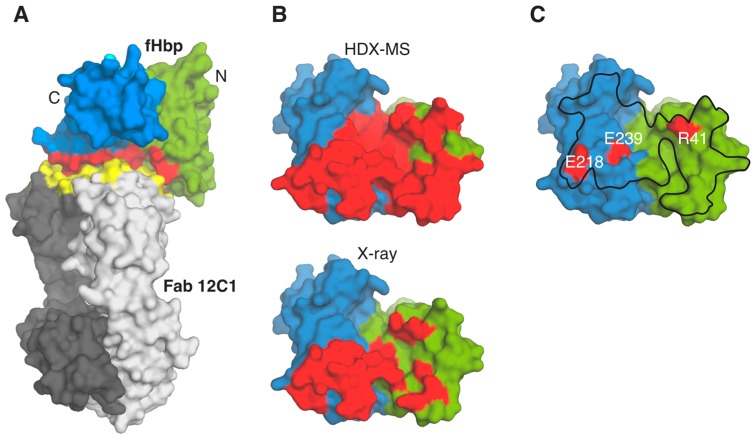
(**A**) The crystal structure of the complex fHbp-Fab 12C1 (pdb 2YPV) is depicted with green/blue surface for N and C termini of fHbp, and light/dark gray for light and heavy chains of Fab 12C1. The epitope and paratope surfaces are colored in red and yellow, respectively; (**B**) Surface representations of fHbp (colored as in panel **A**), allowing comparison of the Fab 12C1 epitope (red patch) as revealed by HDX-MS (**top**) and X-ray crystallography (**bottom**). For clarity, the surface of fHbp only is shown, after re-orientation (~90° about the *Y*-axis) of the view in **A**; (**C**) Surface locations of fHbp residues (red patches, labeled) which when mutated to Alanine inhibit human fH binding. The entire interface of the interaction with fH on the surface of fHbp is outlined with a black line, as revealed previously [41].

Indeed, both EM and HDX-MS have emerged as increasingly powerful tools for mapping conformational epitopes under native conditions, *i.e.*, using full-length folded proteins, not linear peptides or fragments [39]. For example, HDX-MS was used to map the protective epitope of a mAb targeting the meningococcal factor H binding protein (fHbp), a *Bexsero*^®^ antigen, and the results were in close agreement with the crystal structure of the same complex [40] (Figure 2A,B). Analysis of the Fab 12C1/fHbp complex structure *in silico* and subsequent sequence and structure-guided site-directed mutagenesis studies revealed that the variant 1-specific conformational epitope targeted by 12C1 is not dependent on just one or two key residues, but rather is determined by a large discontinuous conformational epitope, which was optimally identified only by protein crystallography. Interestingly, the Fab binding site on the surface of fHbp overlapped significantly with the binding site of human factor H revealed by a previous co-crystal structure [41], and this competition for overlapping surfaces may well contribute to the strong bactericidal efficiency of this mAb [42].

Another example of epitope mapping by hybrid methods combining HDX-MS and protein crystallography was recently reported, describing a bactericidal mAb (33E8) that targets NadA variant 3, and which could not be crystallized (see above) [20]. The Fab 33E8/NadA interaction was mapped by HDX-MS and combined with the homology model of NadA variant 3, built using the crystal structure of variant 5. This study revealed the possible basis for the lack of binding of mAb 33E8 to the second group of NadA variants 4, 5 and 6, since the conservation of the sequences between the two groups within this epitope was relatively low [20] (Figure 1A). Similarly, a recent hybrid method approach using negative stain electron microscopy (NS-EM) and particle reconstruction was performed to map the binding site of a neutralizing mAb on the human cytomegalovirus (HCMV) glycoprotein gH/gL or gH/gL/gO complexes, information which, when coupled with HDX-MS data, and a homology model built using the crystal structure of herpes simplex virus (HSV) glycoprotein gH/gL complex [43], provided insights into the key determinants of the conformational epitope and the 3D architecture of the antibody/antigen complexes [44]. Collectively, these studies indicate that increased efforts to structurally characterize antigen–antibody interfaces, by protein crystallography alone or via hybrid methods, are required to fully understand the antigen recognition by the immune system and can provide insights regarding the mechanism of action of protective or neutralizing mAbs.

Despite various efforts to develop alternatives, protein crystallography remains one of the most powerful techniques allowing fine mapping of epitope–paratope interfaces. A co-crystal structure provides a visually immediate and highly comprehensive definition of the interface. To date there are over 100 non-similar antibody-antigen (*i.e.*, Fab-protein) complex structures deposited in the protein data bank (PDB), providing a wealth of information about molecular recognition by the immune system [45]. Thus, epitope mapping has become one of the most widespread and important applications of protein crystallography in the field of vaccine research.

Nevertheless, protein crystallography has several practical limitations and cannot always provide epitope mapping information within short timelines. For instance: (i) the generation of crystals typically requires relatively large amounts of sample; and (ii) even when sufficient sample is available, there is no guarantee that the antigen–antibody complex will actually produce high-quality crystals. Despite this, the use of Fabs, single-chain variable fragments (scFvs) or single-domain antibodies is an emerging tool to “chaperone” the crystallization of recalcitrant proteins, and therefore it can be anticipated that the probability-of-success when crystallizing antigen-antibody complexes may in fact be higher than that when crystallizing antigens alone, largely due to the solubilizing and/or stabilizing effect of the antibody component and the generation of new regions that can provide crystal packing interfaces [46]. For example, on a “local” scale, complex formation with an antibody might stabilize flexible surface-exposed loops in one of several relevant low-energy conformations, thus overcoming flexibility that might otherwise inhibit productive crystallization. Or alternatively, on a “whole molecule” scale, complex formation might stabilize a large conformationally-heterogeneous protein in a particular state otherwise difficult or impossible to crystallize. An additional benefit is that co-crystallization enables use of the Fab as the molecular replacement search model to provide phasing information during structure determination. The increasing ability to produce high-quality recombinant Fabs for co-crystallization studies will facilitate this approach [36,37,38]—providing more, high-quality “shots on goal” than possible when using only a few Fabs derived from standard hybridoma techniques.

Summarizing, given the value of protein crystallography in the vaccine field, we anticipate a strong expansion of epitope mapping applications in the immediate future, likely to include an increasing proportion of antibody fragments derived directly from individually-cloned human B-cell sequences. Moreover, crystallographic epitope mapping can also potentiate the optimization of therapeutic antibodies and indicate the most appropriate antibody–antigen pairs for diagnostic applications or *in vitro* potency assays designed to monitor the stability of the most relevant components of a vaccine formulation.

## 3. Structure-Based Antigen Design

The ultimate goal of using protein crystallography in vaccine research is to enable the design of novel antigens with enhanced characteristics. This section reviews several notable examples of structure-based antigen designs, some of which introduce and demonstrate promising new approaches, and some of which have now progressed from pre-clinical into clinical trials.

### 3.1. Optimizing the Factor H Binding Protein Antigen of Serogroup B Meningococcus

Meningococcal factor H binding protein (fHbp) binds to human factor H (hfH) and down-regulates complement activation, thus evading complement-mediated killing and promoting bacterial survival [47,48]. Slightly different forms of fHbp are included in both of the recently-licensed serogroup B meningococcal vaccines [10,11]. To date, there are over 800 distinct fHbp amino acid sequences known [49] and they can be classified into three main variant groups, which exhibit 90%–100% sequence identity within the groups, but as little as 63%–85% sequence identity across the groups. The fHbp sequence variation is presumably an immune evasion mechanism that appears responsible for the lack of cross-variant protection afforded by wild-type molecules [47,50,51,52]. Of these antigens, the most well-characterized is fHbp variant 1, for which several structures have been determined [53,54,55]. Crystal structures of representatives from each of the variant groups 1, 2 and 3 have been determined, alone or in complex with hfH [41,54,56] or with a bactericidal mAb specific for fHbp variant 1 [40]. The recently-approved anti-meningococcal vaccines are expected to save many lives and much suffering by preventing invasive meningococcal disease, and yet these current first-generation vaccines are unable to guarantee protection against all possible strains, largely due to the high sequence variability exhibited by MenB surface antigens, especially fHbp. Therefore, structure-based design efforts have targeted two major issues: (i) how to engineer an improved fHbp molecule that combines the entire immunogenic repertoire of all the 3 variant groups into a single broadly-protective antigen; and (ii) how to engineer a factor H nonbinding form of the antigen.

#### 3.1.1. Overcoming Sequence Variability

Structural studies of fHbp variant 1, combined with basic epitope mapping data, suggested that amino acids contributing to the immunogenicity of variant 1 or variants 2 and 3 were located in non-overlapping regions. This intriguing observation of variant-specific epitope patches led to the development of a “chimeric” antigen displaying an immunogenic subset of variant 2 or 3 residues on the variant 1 backbone [57]. In short, using the variant 1 fHbp as a scaffold, patches of residues from variants 2 and 3 were grafted onto the protein surface (replacing variant 1 residues). Each patch encompassed approximately 900–2000 Å^2^ of surface area, and approximately 50 different proteins were designed and tested, in order to fully explore the strategy. The approach was successful in generating an antigen able to elicit more broadly cross-protective antibody responses in pre-clinical studies in mice [57], as reviewed previously [58,59]. Notably, crystal structure determination of the most effective engineered chimeric fHbp protein, which contained over 20 simultaneous surface-exposed point mutations, confirmed that the surface had been successfully manipulated to display an epitope bearing the characteristics of the variants 2 and 3 groups, but without affecting the overall fold of the protein, thus leaving the vast majority of variant 1 epitopes undisturbed.

#### 3.1.2. Elimination of Undesirable Function

The structure of the fHbp:fH complex provided insights on the potential interference of fH binding by fHbp with immune recognition or antibody binding when used as a vaccine antigen [41]. Since the affinity for fH is very high and the site of interaction between fHbp and fH quite extensive, the concern arose that a functional fHbp, able to bind fH, could have a reduced number of epitopes available for recognition by antibodies, as these would be obscured by the bound fH. Thus, the hypothesis that the structure of the complex could be used to design an engineered fH nonbinding antigen was advanced, considering that this would make a superior antigen, with higher immunogenicity as the resultant antibody responses would be directed also at epitopes in or near the now exposed fH binding site, resulting in greater complement-mediated serum bactericidal activity (Figure 2C).

First, two double mutants of fHbp were designed (residues R341A/H337A and E283A/E304A, the latter subsequently renumbered to E218A/E239A [60]) and studied by surface plasmon resonance (SPR), revealing the expected loss-of-function [41]. Later, it was shown that the mutant E218A/E239A was less immunogenic than wild-type fHbp, as it elicited up to 20-fold lower serum bactericidal titers than those elicited by a wild-type fHbp [60]. Subsequent studies characterized the binding and immunogenicity of other fH nonbinding mutants [56,60,61]. Structure-based design was also performed by Granoff and co-workers to remove a charged hydrogen-bond with fH mediated by a surface-exposed fHbp arginine [61]. This Arg-41-Ser mutation resulted in no detectable fH binding and a nearly twenty-fold higher protective antibody response in a mouse model [62]. This design was also supported by previous knowledge of fHbp epitopes that elicit bactericidal antibodies, allowing confident prediction that the Arg-41-Ser substitution would have no effect on serum bactericidal antibody responses to the mutant fHbp antigen, as subsequently confirmed [61]. The study of key fHbp amino acids necessary for high affinity fH interactions was also extended to other fHbp variant families, revealing how different variants engage fH in distinct ways, though all using the same molecular region overall [56]. In addition, in this same study, the crystal structure of the double mutant E218A/E239A was determined, showing that the only detectable change compared to WT was the loss of the side chains of E283 and E304, thus the overall fold was conserved [56]. More recently, Tang and co-workers showed how two nonfunctional v3 fHbps retain their immunogenicity, and although these mutants (T286A and E313A) possess a marked reduction in affinity for fH, their folding was apparently not affected by the mutations [63]. Although some creative transgenic mouse models that approximate the expression of human fH have shown the benefits of these nonbinding fHbps [56,61], it is clear that rationally-designed fHbp antigens will need to be tested and compared in human clinical trials, in order to estimate the impacts and potential benefits of the loss of fH-binding ability.

### 3.2. Nonbinding Mutants of Transferrin Binding Protein B

The concept of engineering non-functional forms of an antigen that do not bind to the natural ligand has also been recently applied for a second bacterial antigen. Mammalian host transferrin (Tf) is used as an iron source by several Gram-negative bacterial pathogens. The surface-exposed Tf binding protein B (TbpB) mediates interaction with Tf [64,65], and in pathogenic *Neisseria* TbpB is thought to orchestrate the “piracy” of the iron cargo from human Tf [66]. Consequently, TbpB is a potential antigen for human or animal vaccines. However, data suggested that upon immunization the formation of the TbpB/Tf complex might mask important epitopes and thereby inhibit generation of the optimal immune response against TbpB. Therefore, TbpB point-mutants with strongly reduced ability to bind Tf were designed based on the crystal structures of TbpB/Tf complexes, combined with insights from homology modelling [67]. In pre-clinical tests in a pig model, a mutant *Haemophilus parasuis* TbpB was shown to induce enhanced immune responses and provide superior protection. These studies further indicated that structure-based strategies can be a powerful way to design “nonbinding” antigens with improved pre-clinical performance.

### 3.3. Multiple Protein F-Based Strategies for a Respiratory Syncytial Virus Vaccine

RSV is the most important viral cause of severe respiratory tract disease in children worldwide [68]. RSV accounts for over 6% of deaths in infants from 1 to 12 months old and is thus a leading viral cause of childhood death [69] and also affects elderly and immunocompromised adults [70]. Although there is a therapeutic humanized monoclonal antibody (palivizumab, named *Synagis*^®^) licensed by the FDA for prophylactic use in children at high risk, and which reduces the incidence of severe disease [71], there is currently no RSV vaccine available, despite over 40 years of targeted research. However, there are now several clinical trials of candidate RSV vaccines ongoing, and there is eager anticipation that these efforts will deliver a much-needed vaccine in the next five to ten years [72,73]. The vast majority of research into RSV candidate antigens has focused on the membrane-anchored fusion glycoprotein F, a highly-conserved target of neutralizing antibodies [74]. Although there is encouraging clinical evidence that RSV F-specific antibodies (including palivizumab) can protect against disease, the development of an F protein antigen as a vaccine candidate has been hampered by several factors, including the biochemical challenge that F has an intrinsic tendency to undergo large conformational changes, a functional requirement typical of viral fusion glycoproteins for mediating viral and cellular membrane fusion, as reviewed previously [75]. The following sections provide three distinct examples of how protein crystallography has provided structural insights that have overcome obstacles in the research and development pathway, thus driving the design of novel F-based antigens, each of which shows promise as a future vaccine antigen.

#### 3.3.1. Rational Engineering of a Soluble, Stable and Homogeneous Post-Fusion F

The RSV F protein forms large (>150 kDa) trimeric structures anchored on the outer face of the virion membrane. Electron cryotomography and negative-stain electron microscopy revealed that F exhibits two main forms [76,77], now termed the pre-fusion and post-fusion F forms. When produced recombinantly, pre-fusion F is only “metastable”, and readily converts to the post-fusion form which, however, tends to aggregate via an exposed hydrophobic fusion peptide [78], thus rendering it challenging for development as a vaccine antigen. By sequence- and structure-based modelling using homologous F protein templates from another paramyxoviridae (parainfluenza PIV5) F protein structure, a novel non-aggregating and highly-stable form of RSV F was designed via removal of the fusion peptide, the transmembrane region and the cytoplasmic domain. The crystal structure of this substantially complete form of post-fusion F was determined [79], and revealed a stable trimer which displayed the key neutralizing antibody binding site of palivizumab and motavizumab (an affinity-enhanced derivative) [80]. The presence of these protective epitopes, and the mAb 101F epitope, which had been previously defined by co-crystal structures of the motavizumab and 101F Fabs in complex with target RSV epitope peptides [81,82], were confirmed independently in a second similar crystal structure determination of post-fusion F [83]. Together, these two structures revealed the molecular basis for the unexpectedly high immunogenicity of the post-fusion F antigen. The engineered F antigen molecule was readily prepared in a homogeneous, stable and reproducible format and was found to elicit high titers of neutralizing antibodies in mice or cotton rat animal models [79]. Clinical trials are ongoing using post-fusion versions of the F antigen.

#### 3.3.2. An Antibody-Dependent Approach to Design and Engineer Pre-Fusion F

**Figure 3:**
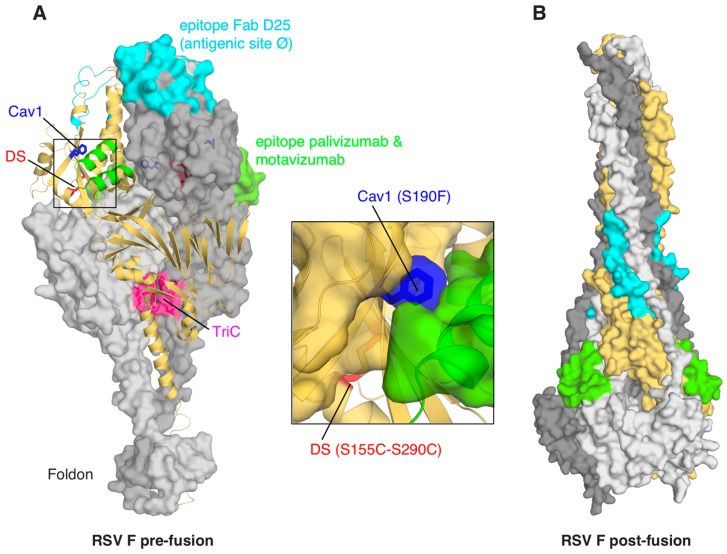
(**A**) Stabilized respiratory syncytial virus (RSV) F pre-fusion (pdb 4MMV) is shown as light and dark surfaces for two chains, and as yellow cartoon for the third chain of the trimer. Sites that were mutated to stabilize the pre-fusion configuration are colored in blue, green, and pink, for the S190F-V207L pair (Cav1), the S155C-S290C double mutant (DS), and the D486H-E487Q-F488W-D489H mutant (TriC), respectively [86]. Known epitope surfaces for Fabs D25, and for palivizumab and motavizumab, are colored in cyan and green, respectively. A zoomed view of the region of DS and Cav1 mutants (central box) provides details of the cavity-filling mutation S190F and of the introduction of the disulfide bridge C155-C290; (**B**) Post-fusion RSV F (pdb 3RKI) [79] is shown as surface, color-coded as in panel **A**.

Significant efforts have also been made to harness the vaccine potential of the more elusive “metastable” pre-fusion F antigen. Conceptually, the pre-fusion F conformation would be a better vaccine target as it exposes all the functional sites and neutralizing epitopes present on virion F. Although engineered post-fusion F can elicit high titers of neutralizing antibodies in animal models [79], a subsequent report demonstrated that antibodies specific for the pre-fusion F form account for most of the neutralizing activity of human sera from seropositive subjects [84]. It thus appeared that some critical neutralizing mAb binding sites were absent in the post-fusion F form and consequently attempts to design a stable pre-fusion F antigen intensified. Important “turning points” that ultimately enabled informed antigen design were the discoveries of a few new anti-F neutralizing mAbs (mouse 5C4, human D25 and AM22) with the unique property of not recognizing a stabilized post-fusion F form. These mAbs were used for structural studies to trap the F molecule in its pre-fusion state. Crucially, after co-expression and co-purification, the crystal structure of Fab D25 bound to RSV F in the pre-fusion conformation was determined [85]. Although the structure revealed that the palivizumab and motavizumab epitopes were well exposed in pre-fusion F, there was a dramatic overall change in conformation (Figure 3). Analysis of the epitope–paratope interface in this complex explained why D25 does not bind to post-fusion F and thus the crystal structure provided mechanistic insights, suggesting that D25 neutralizes RSV by restraining F in the pre-fusion state. The epitope recognized by D25, site Ø, which is also the target of 5C4 and AM22, is on the most exposed apex of F, which may underlie the higher effectiveness of neutralizing antibodies against this region, despite having a binding affinity similar to that of motavizumab. These structural studies led to the proposal that F antigens stabilized in the pre-fusion conformation may further improve the immunogenicity of this molecule. Indeed, stabilization of the trimer by addition of a trimerization tag (a foldon) replacing the transmembrane region, structure-based insertion of hydrophobic packing mutations and judicious insertion of a novel disulfide bond, forms the basis of a leading pre-fusion F candidate antigen (Figure 3). Of note, similarly to the iterative approach of structure-based design used for the development of high-affinity drugs, the authors developed a method to screen hundreds of structure-guided mutations to identify those resulting in protein stabilization and favorable expression levels. Most importantly, in mouse and nonhuman primate animal models, a stabilized pre-fusion F molecule elicited RSV-specific neutralizing titers significantly greater than those elicited by a post-fusion F protein and well above the protective threshold [86].

#### 3.3.3. Epitope-Focused Vaccine Design to Target a Neutralizing Epitope on the F Antigen

As introduced above, development of an RSV vaccine based on the wild-type F protein has been hampered by the large conformational changes it undergoes, and its relatively poor behavior in solution. Consequently, alternative approaches to F-based antigen design were sought, based on the concept of using selected scaffold proteins to display structured peptide fragments representing neutralizing epitopes of F. This goal was greatly facilitated by determination of the crystal structure of the RSV-neutralizing motavizumab Fab in complex with its peptidic epitope from the F protein, wherein motavizumab was observed bound to its 24 residue peptide target which adopted a helix-turn-helix conformation [82]. However, initial structure-based design efforts to generate RSV epitope-scaffold immunogens were only partially successful, insofar as they induced structure-specific anti-F antibodies but without RSV-neutralizing activity [87]. Subsequently, a major proof-of-principle that epitope-scaffold immunization can re-elicit neutralizing antibodies against a pre-defined target epitope was obtained by developing new computational methods to design or optimize novel scaffolds tailored for the motavizumab epitope structure [88]. Briefly, a novel scaffold with robust biophysical, structural and antigenic properties was designed to faithfully display the motavizumab helix-turn-helix epitope. Achievement of the design objective was confirmed by crystal structure determination of an epitope-scaffold alone or in complex with the motavizumab Fab, which revealed a high degree of epitope mimicry. Further, several epitope-scaffolds were recognized by sera obtained from RSV-seropositive humans, confirming that a clinically-relevant epitope conformation was presented. Finally, immunization of rhesus macaques with three slightly different motavizumab epitope-scaffolds alone, or one epitope-scaffold construct mounted in multiple copies on a hepatitis B core antigen-derived virus-like particle (VLP), were sufficient to induce F-binding antibodies in all animals. Notably, in at least half of the epitope-scaffold VLP immunized animals, the elicited RSV-neutralizing activity was comparable to the neutralization titers induced by natural human infection.

### 3.4. Human Immunodeficiency Virus (HIV)—The Ultimate Challenge?

HIV affects more than 30 million people worldwide, killing ~2 million people per year and it remains a major global public health threat [89]. There is currently no cure for HIV infection, though it may be controlled with effective antiretroviral treatment. However, in many countries, the cost of antiretroviral therapy is prohibitive. Prevention of HIV transmission is certainly the long term global solution for the HIV pandemic and vaccination is likely the most sustainable mechanism to achieve this goal.

The envelope glycoprotein (Env) is the only target for neutralizing antibodies of HIV-1. Env is responsible for fusion between the viral and cell surface membranes and allows entry of HIV into the target host cell. Env is produced as the gp160 precursor which is cleaved by furin to generate a heterodimer composed of gp120 and gp41, three copies of which form the trimeric Env spike [90]. HIV is highly successful in thwarting the immune response, in part due to its high mutation rate that results in high sequence diversity of the spike, thus hindering the development of potently and broadly neutralizing antibodies.

Over the last ~30 years, HIV vaccine design programs have attempted various strategies to generate humoral or cellular immunity, or, more recently, both. The largest proof of the feasibility of a vaccine preventing HIV infection came in the RV144 Phase 3 trial in Thailand, which used a recombinant canarypox prime expressing gp120_TM and a bivalent gp120 (subtype B, MN and subtype A/E, A244) protein boost [91]. This study showed modest efficacy over three years of follow-up. Subsequent intensive evaluations have shown that V1V2 (variable regions 1 and 2) antibodies may be associated with less risk of subsequent HIV infection [92]. These findings energized the field and suggested that HIV vaccination may work and new human trials are now being conducted. A comprehensive survey of the vast body of literature describing the search for HIV vaccine antigens is beyond the scope of this review; the interested reader is invited to consult other recent and more extensive HIV-dedicated resources (as excellent examples, see [93,94]). Nevertheless, to serve our particular objective in highlighting the roles of protein crystallography in vaccine research, the following sections describe a number of innovative approaches that have been developed in the past several years aiming to generate an effective HIV vaccine antigen. Indeed, the development of a protective HIV vaccine remains one of the greatest challenges facing vaccinologists today.

#### 3.4.1. Scaffold-Based and Multi-Copy Approaches in the Design of HIV Antigens

Some of the earliest breakthroughs in HIV structural biology were achieved over 15 years ago by co-crystallization of gp120 sub-domains with deletions in the variable regions together with soluble CD4 (the host receptor) and Fab fragments [95]. Several different structurally- and computationally-based HIV antigen design projects have followed, mainly focused on gp120 and portions of gp41, and some promising results have been obtained. For example, scaffolds displaying known HIV gp120 and gp41 epitopes in a more stable fashion than in their native context have been generated. Namely, starting from antibody/epitope co-crystal structures, Schief and co-workers described computational methods for the design of optimized epitope scaffolds that showed high conformational stability and were therefore good candidates for the presentation of known structural epitopes to the immune system [96]. In a similar study, the same group performed the grafting of a discontinuous gp120 epitope onto a scaffold protein unrelated to gp120, with the appropriate retention of structural and antigenic properties [97]. Further, Kwong and co-workers grafted neutralizing epitopes of HIV-1 gp41 onto a protein backbone scaffold more stable than gp41 itself [98]. Continued efforts to identify and characterize antibodies able to broadly cross-neutralize many strains of HIV are likely to further fuel this epitope-based approach to vaccine design.

In addition to the structure-based computational design of conformationally-correct epitope scaffolds, different groups have used the structures of gp120 bound to the CD4-receptor as the starting point to design mutations that lock full-length or truncated forms of gp120 in the CD4-bound conformation [99,100]. When the stabilized molecules were used to immunize small animals, they were found to elicit a higher proportion of antibodies targeting the conserved CD4 and co-receptor binding sites than the wild-type antigen. Recently, an elegant approach to target the germline B-cell precursor of an affinity-matured broadly-neutralizing antibody has been developed [101]. The authors of this study designed an HIV gp120 outer-domain immunogen that bound to VRC01-class broadly neutralizing antibodies and their germline precursors. Of note, when presented on a lumazine synthase self-assembling nanoparticle, this immunogen was able to activate germline and mature VRC01-class B-cells [101]. This approach may be particularly useful for the elicitation of rare antibodies targeting specific neutralizing epitopes.

#### 3.4.2. Structure Determinations of the HIV Envelope Glycoprotein (Env) Trimer

Despite being a great focus of attention, Env has been highly resistant to structural characterization, in particular via crystallization, mainly due to its heterogeneous metastable nature, conformational heterogeneity and extensive glycosylation. Over the last 12–18 months, major advances have been achieved, using both X-ray crystallography and cryo-electron microscopy (cryo-EM) to structurally characterize the Env trimer and Env–antibody or Env–receptor interactions. The crystallographic studies first required production of a soluble, stable, cleaved form of the Env trimer, which was achieved by inserting a covalent disulfide bridge between gp120 and gp41, coupled with a point mutation to generate a more stable gp41 trimer (Ile to Pro, in the N-terminal heptad repeat). Ultimately, several protocol adjustments were combined to obtain a well-behaving form of Env, structurally and antigenically similar to the native form, and suitable for crystallization. The crystal structure of an HIV-1 Env trimer was first determined at ~4.7 Å resolution [102], and subsequently at ~3.5 Å resolution [103], revealing a stem-and-head spike structure, with gp41 in the pre-fusion conformation and the trimer apex stabilized by inter-protomer interactions of the gp120 V1, V2 and V3 variable loops. The mature closed-state pre-fusion structure, the target of most neutralizing antibodies, was shown to be covered with N-linked glycosylations (25–30 per gp120-gp41 protomer) and rich in sequence-variable regions, major hallmarks of immune evasion strategies [103]. A very similar Env structure was simultaneously determined by cryo-EM at 5.8 Å resolution [104]; both the X-ray and EM structures revealed complexes with broadly-neutralizing antibody Fab fragments, and thus provided insights into protective epitopes on Env, which are potential sites of vulnerability to antibodies and ideal platforms for continued structure-based design of candidate vaccine antigens. Early efforts have been made to convert this sort of engineered Env construct into next-generation antigens [105]. However, it remains to be seen whether these most recent structural insights will ultimately lead to the design and development of an effective vaccine antigen for clinical trials.

## 4. Enabling Technology for Protein Crystallography in Vaccine Research

During the last ~80 years, fueled by technological advances crystallography has made dramatic progress and a revolutionary expansion, becoming integral to modern biology, medicine, and drug discovery [106,107]. The determination of macromolecular structures using X-ray diffraction involves many technologies such as molecular biology, bioinformatics, and more generally physical sciences. Progress in recombinant DNA technology, crystallization methods, synchrotrons, computing, phasing and refinement algorithms, drove the strong expansion of crystallography, which resulted in more than 100,000 structures of biological macromolecules and macromolecular assemblies being deposited in the Protein Data Bank as of 2014 [108]. Although structure determination by protein crystallography is still not a high-throughput discipline, several recent advances have increased throughput and the probability-of-success of crystal structure determination, often stimulated by the observations that have emerged from large-scale structural genomics initiatives over the last two decades [109].

### 4.1. A Short Introduction to X-ray Crystallography of Proteins

The birth of protein crystallography can be traced back to the observation by Bernal and Crowfoot of the first X-ray diffraction pattern from crystals of pepsin, which revealed that proteins had an ordered three-dimensional structure [110]. The first protein structure to be determined was that of myoglobin [111,112], followed soon after by those of haemoglobin and lysozyme, the first structure of an enzyme, in 1960 [113] and 1962 [114,115]. While a comprehensive and recent overview of the pipeline of protein crystallography and of its developments can be found elsewhere [116], here we provide a concise summary of the main steps and technical challenges of the method.

In order to solve the three-dimensional structure of a protein by X-ray crystallography, the first prerequisite is to crystallize the macromolecule of interest. Crystals are made of billions of the same molecules in an ordered array, and this arrangement allows the magnification of the diffraction signal that is essential to overcome the weak, and not measurable, diffraction from a single molecule. The crystalline state confers to each molecule the same scattering properties. However, since proteins and nucleic acids do not naturally arrange into a regular and periodic manner as is typical of crystals, crystallization is often the rate-limiting step in protein crystallography. Several strategies for overcoming the intrinsic difficulties of the crystallization of biological macromolecules have been devised (see below). Once highly-ordered and well-diffracting crystals have been obtained, data collection experiments are performed, where the diffraction pattern of the crystals is recorded by placing them into an X-ray beam. When X-rays strike the crystal, the atoms it contains will produce scattered X-ray waves, and the energy (or amplitudes) and positions of these scattered waves are recorded during data collection. In theory, by summing all the scattered waves the structure of the macromolecule can be solved. However, the origin of each wave must first be determined, which corresponds to observing the time of arrival of the X-ray peaks scattered in different orientations. This information is not directly accessible or experimentally measurable, and this is known as the phase problem, which is another major obstacle in the structure determination by X-ray crystallography. Different computational and experimental methods to solve the phase problem are available today, and their development is discussed below. Once phases have been assigned to each scattered wave, their summation in three dimensions generates the electron density distribution of the molecule of the crystal. This process is performed by use of the Fourier transformation, which requires (1) the structure factor amplitudes measured during data collection; and (2) their relative phase angle. The initial electron density obtained upon solving the structure is subsequently used to trace or fit models of the crystallized molecule, thus providing the first picture of the structure of the protein. The coordinates of the model structure are subsequently subjected to refinement, to improve their overall quality by tweaking calculated model parameters such as atom positions and displacement (also called temperature or B-factors), until they best describe the experimentally observed data. Refinement will determine the final quality of a crystal structure, which also depends on the resolution of the X-ray diffraction data. A typical crystallographic dataset can enable structure determination in a resolution range from high (~1 Å) to low (~3.5 Å), providing sufficient detail to observe the positions of all (non-Hydrogen) backbone and side chain atoms, or the shape of the molecule and the secondary structure elements, respectively. There is currently no other technique that can routinely deliver such highly-detailed and precise atomic-level information, and such data has over the last 50 years provided innumerable structural and functional insights, enabled many protein engineering efforts, as well as the design of small-molecule inhibitors now available as pharmaceutical agents to treat a variety of medical conditions.

### 4.2. Advances in Protein Crystallization

The production of high-quality crystals for X-ray crystallography is famously a major bottleneck in structure determination. Although there is no apparent correlation between crystallization propensity and protein structure, nor “magic bullets” for the production of good crystals, a variety of methods to aid crystallization exists and these have been extensively reviewed [117,118,119]. Structural genomics initiatives have been a major driving force for the automation and progress of large-scale expression, purification of recombinant proteins, and protein crystallization methods [120,121,122,123]. A list of advances that facilitate protein crystallization can be attempted by dividing these into two groups, based on how time- and labor-intensive they are. Group 1 includes those that involve or require cloning strategies and were enabled or facilitated mainly by the development of high-throughput recombinant methods and new cloning vectors; Group 2 includes more general and simple rescue tools or strategies to obtain better crystals or to improve existing sub-optimal crystals.

#### 4.2.1. Group 1

- High-throughput domain-hunting strategies to search for optimal expression constructs for the bacterial production of difficult target proteins [124,125];
- Mutations to stabilize the target protein [126,127];
- Surface entropy reduction (SER): mutation of surface residues to create patches of low entropy that can preferentially mediate crystal contacts [128];
- Sequence homolog screenings: sequence variability between homolog proteins, if localized on the surface, can favor better packing and crystallization [21];
- Fusion proteins: highly crystallizable proteins (*i.e.*, T4 lysozyme) covalently fused to disordered regions of the target protein, well-known to aid crystallization of GPCRs [129,130].

#### 4.2.2. Group 2

- Binding partners (or chaperone-assisted crystallography): Fab fragments, single-domain antibodies, synthetic antibodies, and more general substrates, may reduce conformational freedom of the target protein and thus enhance propensity to form the ordered lattice required for crystallization [118,131,132];
- *In situ* proteolysis: the addition of trace amounts of proteases in crystallization trials can help to enzymatically eliminate flexible or disordered regions that might hinder crystallization [133];
- Seeding: separating crystal nucleation and growth, use of seeds from microcrystalline material or precipitate to streak in a freshly prepared protein solution [134,135];
- Reductive methylation: targets lysine residues, modifying their primary free amines to tertiary amines, and thus likely decreasing disorder on the protein surface [136,137].

In addition to the developments above, a considerable amount of crystallization data has been amassed, and this is now being used to develop statistical analyses for predictive strategies for crystallization [138,139,140]. Also, progress in instrumentation for crystallization droplets imaging now allows further developments towards automatic ranking and classification of the droplets [141].

### 4.3. Advances Facilitating the Determination of Crystal Structures

As for crystallization methods, technological advances and structural genomics initiatives have largely powered progress in methods for protein structure determination and refinement, moving the field towards automatic crystallographic structure solution tools. Astonishing progress has been accomplished in all fields from synchrotron radiation/X-ray diffraction data collection, to phasing and structure refinement.

Synchrotron radiation is now the main source for X-ray diffraction, and it virtually entirely replaced sealed-tube and rotating-anode generators that were common in crystallographic laboratories until the 1990s. Compared to a rotating-anode source, the increase of the X-ray flux of a third-generation synchrotron facility is of 20 million times [142]. The potential of synchrotron radiation application for crystallography was first recognized in the late 1940s, and enabling technologies that allowed the construction of synchrotrons with appropriate high energies started to develop in the 1960s. The first use of synchrotron radiation for protein crystallography can be traced back to the 1980s. In the early 2000s, a new hybrid pixel X-ray detector (PILATUS) was introduced, which is now in standard user operation on an increasing number of beam lines [143]. The introduction of the PILATUS, which operates in single-photon counting mode and possesses a fast readout time and the absence of readout noise, profoundly changed data-collection strategies [144]. Among the most recent developments of synchrotron facilities are automation, remote user access, and industrial service provision [145,146,147]. More recently, the advent of free-electron lasers (FELs), which deliver extremely intense femtosecond X-ray pulses, allowed the development of serial femtosecond crystallography (SFX) [148]. SFX promises to overcome two major limitations of protein crystallography, small crystal size and radiation damage, and examples of applications have been published recently [149,150,151].

The availability of tunable X-rays from synchrotron sources in the 1990s allowed the implementation of the multi-wavelength anomalous dispersion (MAD) phasing method [152]. At the time, the commonly used phasing method was multiple isomorphous replacement (MIR), where heavy-metal ions were incorporated, mostly by time- and labor-intensive soaking experiments into native crystals [153]. Measurements of the perturbation of the diffraction pattern of heavy-metal soaked crystals compared to native crystals were then used to obtain information on the possible values of the phase angle, with the critical caveat that measurements had to be performed with very high accuracy, and native and derivative crystals needed to be isomorphous [154]. The introduction of MAD overcame these obstacles, as only one single crystal containing atoms capable of anomalous scattering (commonly seleno-methionine labelled proteins) was needed. However, to perform a MAD experiment required the collection of a number of X-ray wavelengths, and combined with the power of synchrotrons, a MAD experiment potentially induced severe radiation damage that could often compromise the measurement of the anomalous signal itself [155]. Later, as more high-throughput methods for structure determination were needed, as well as methods that would induce less or no radiation damage, single anomalous dispersion (SAD) was developed [156,157]. Being faster and easier to perform, phasing by SAD has been successfully adapted in high-throughput pipelines [158,159]. Other phasing methods that have gained popularity with the advent of high-throughput crystallography projects are those that exploit the soakings of halides in native crystals [160]. These are particularly advantageous in cases where a protein does not bind heavy-metal atoms or cannot be prepared as a seleno-methionine variant. Halide anions, such as bromide or iodide, have been shown to be easily incorporated into the crystal solvent regions around protein molecules, and as such they allow measuring an anomalous signal, thus providing phasing power [161].

Due to the increasing number of protein structures available today in the PDB, molecular replacement (MR) is now the most widely used phasing method [162]. The method was introduced in the 1960s; it is based on the availability of a suitable related model (the template) and consists of a trial-and-error search where all possible orientations and positions of the template model are explored in the unit cell of the unknown crystal target [163]. Perhaps, the most critical step for the success of MR is the selection and modification of the template model structure, which is usually made based on sequence homology. Progress in bioinformatics and sequence manipulation software, and the availability of many sequences and structures in the databases allow accurate multiple alignments that can aid in the selection of an optimal template for MR [164]. In addition, several automated software tools that streamline the process of finding homologs and generating a suitable template, as well as running the MR searches using different softwares such as Phaser [165], and Molrep [166], and performing the initial refinement, are now available [167,168,169]. The automation of crystallographic structure solution has also seen tremendous progress over the last decade with the specific development of many new software tools [170,171] and user support [172].

## 5. Conclusions and Outlook

Here, we have reviewed how protein crystallography can play a key role in vaccine research and development processes. Once the potentially-useful antigens of a pathogen have been identified, structural biology can have an impact on several stages of product development. The value of structure-based antigen design can be perceived at several stages along the pathway, for example (i) to eliminate undesirable regions of the antigen (catalytically-active sites, or immunodominant decoy epitopes); (ii) to stabilize the antigen in the most beneficial conformation; (iii) to guide presentation of the most relevant antigenic epitopes, preferably with an orientation tailored to elicit a targeted immune response; (iv) to assemble the antigen in multi-valent arrays for enhanced immunogenicity; (v) to identify ideally-located sites for molecular conjugation, either of other protein antigens (thus creating larger polypeptides with multiple antigen features) or of smaller molecules, to enable site-specific labelling with antigenic oligosaccharides or immune-potentiator compounds. During later stages of vaccine development, structure-based design can also be used to build-in biophysical or biochemical features that enhance the productivity, stability and safety of the vaccine antigens.

Here, we initially focused on some of the contributions made by X-ray crystallography in the characterization of protein antigens. With emerging technical advances, we anticipate that several other techniques may also play growing roles in this field in the very near future. In particular, cryo-EM has become the preferred method to study icosahedral viruses (featuring high symmetry) [173,174,175] and has already shown promise in the characterization of large antigens and membrane proteins with structures now determined at atomic resolution [176,177]. In particular, EM is an excellent method to rapidly study antibody–antigen interactions at moderate resolution and with low sample quantity requirements [44,178] and can be combined with crystallographic data to provide detailed structural information. This hybrid approach may be particularly powerful when considering the increasing ease with which antibodies can be cloned from human B-cells and can be recombinantly produced and purified with moderate throughput. Another area, partly introduced above, is the use of HDX-MS for the rapid characterization of protein–protein interfaces, with obvious application to antigen–antibody complexes. In contrast with crystallographic or cryo-EM approaches, HDX-MS also holds the intriguing possibility that such studies might be applicable to polyclonal antibody–antigen mixtures [179]. Further, the addition of electron transfer dissociation (ETD) technology to the HDX-MS approach is likely to enable improvements in the resolution of structural MS-based epitope mapping studies [180].

Collectively, these discussions point to the growing intercalation of the fields of human immunology and structural biology. We expect X-ray crystallography to continue to deliver the core information needed for precision design of optimized antigens. However, we also eagerly anticipate the continued development and ensuing contributions of other structural technologies and increasing computational power, thus potentiating the tool-kit available when attempting to address the urgent need for the development of antigen components of novel vaccines designed to control or eliminate infectious disease.

## Abbreviations

Context-relevant definitions of abbreviations or professional terms used herein, listed in the order in which they appear in the manuscript:

Reverse vaccinology: a genomic approach for the sequence-based computational identification of proteins for experimental testing as candidate vaccine antigens.

mAb: a monoclonal antibody, a protein produced by the clones of a single hybrid cell obtained from the fusion of a B cell with a tumor cell (or, more recently, after cloning the relevant gene fragments from a mammalian B cell followed by recombinant expression) and with binding targeting a specific antigen; those discussed herein are of the IgG class, and each IgG is composed of four chains: two identical heavy chains (each of ~50 kDa) and two identical light chains (each of ~25 kDa), such that each mAb is ~150 kDa. There is significant flexibility in several regions of a mAb, such that they are highly recalcitrant to crystallization.

Fab: the fragment antigen-binding of a mAb, encompassing the antigen combining site. A Fab is a heterodimer, composed of one entire light chain (variable-light and constant-light regions, VL and CL) and part of one heavy chain (the variable-heavy and constant-heavy-1 regions, VH and CH1). Fabs are much less flexible than mAbs, and consequently are more likely to crystallize than mAbs.

Meningitis: a potentially-fatal inflammation of the meninges, those membranes surrounding the brain and spinal cord, usually caused by pathogenic infections.

Glyco-conjugate: a conjugate of a polysaccharide or oligosaccharide component derived from the cultured target pathogen and covalently linked to a carrier protein which enhances the immune response against the saccharide (the most common carriers are inactive toxins, such as CRM197, diphtheria toxoid, or tetanus toxoid).

Structural Vaccinology: the use of structural biology data to drive the design and/or optimization of vaccine antigens.

Crystal packing interfaces: typically relatively small interfaces needed to mediate the protein-protein contacts that result in assembly of the ordered array of molecules that defines a protein crystal. Such interfaces are not necessarily physiologically-relevant, *i.e.*, do not necessarily correspond to intermolecular contacts observed in solution under native conditions.

Serum bactericidal activity (SBA): serum bactericidal antibodies have been accepted as the surrogate for protection against meningococcus. In an SBA assay, bacteria are killed based on the cumulative action of specific antibodies in the serum directed against antigens on the bacteria. The amount of serum required for efficient bactericidal killing thus gives an indication of the functionality of the antibodies raised by the antigen.

Foldon: a tag of approximately 27 amino acids that promotes trimerization, derived from the trimeric C-terminal domain of the protein fibritin of the T4 bacteriophage.

Epitope mapping: the identification of the amino acid residues (or atoms) of an antigen that are contacted by a specific antibody.

Epitope grafting: the use of genetic engineering to insert epitope residues of one antigen into the amino sequence of a second (scaffold) antigen.

Paratope: the amino acid residues (or atoms) of an antibody that are contacted by a specific antigen.

Surface entropy reduction: the use of genetic engineering to replace amino acids (typically Arg, Lys, Glu, Asp) exposed on a recombinant protein surface with smaller, less flexible amino acids (typically Alanine) such that the protein surface exhibits lower entropy.

## References

[1] Plotkin S. (2014). History of vaccination. Proc. Natl. Acad. Sci. USA.

[2] Whitney C.G., Zhou F., Singleton J., Schuchat A. (2014). Benefits from immunization during the vaccines for children program era—United States, 1994–2013. Morb. Mortal. Wkly. Rep..

[3] Delany I., Rappuoli R., de Gregorio E. (2014). Vaccines for the 21st century. EMBO Mol. Med..

[4] Rappuoli R. (2001). Reverse vaccinology, a genome-based approach to vaccine development. Vaccine.

[5] Pizza M., Scarlato V., Masignani V., Giuliani M.M., Arico B., Comanducci M., Jennings G.T., Baldi L., Bartolini E., Capecchi B. (2000). Identification of vaccine candidates against serogroup B meningococcus by whole-genome sequencing. Science.

[6] Giuliani M.M., Adu-Bobie J., Comanducci M., Arico B., Savino S., Santini L., Brunelli B., Bambini S., Biolchi A., Capecchi B. (2006). A universal vaccine for serogroup B meningococcus. Proc. Natl. Acad. Sci. USA.

[7] Pace D., Pollard A.J. (2012). Meningococcal disease: Clinical presentation and sequelae. Vaccine.

[8] Pace D. (2009). Quadrivalent meningococcal ACYW-135 glycoconjugate vaccine for broader protection from infancy. Expert Rev. Vaccines.

[9] Finne J., Bitter-Suermann D., Goridis C., Finne U. (1987). An IgG monoclonal antibody to group B meningococci cross-reacts with developmentally regulated polysialic acid units of glycoproteins in neural and extraneural tissues. J. Immunol..

[10] O’Ryan M., Stoddard J., Toneatto D., Wassil J., Dull P.M. (2014). A multi-component meningococcal serogroup B vaccine (4CMenB): The clinical development program. Drugs.

[11] Zlotnick G.W., Jones T.R., Liberator P., Hao L., Harris S., McNeil L.K., Zhu D., Perez J., Eiden J., Jansen K.U. (2015). The discovery and development of a novel vaccine to protect against *Neisseria meningitidis* serogroup B disease. Hum. Vaccines Immunother..

[12] Serruto D., Bottomley M.J., Ram S., Giuliani M.M., Rappuoli R. (2012). The new multicomponent vaccine against meningococcal serogroup B, 4CMenB: Immunological, functional and structural characterization of the antigens. Vaccine.

[13] Łyskowski A., Leo J.C., Goldman A. (2011). Structure and Biology of Trimeric Autotransporter Adhesins.

[14] Capecchi B., Adu-Bobie J., di Marcello F., Ciucchi L., Masignani V., Taddei A., Rappuoli R., Pizza M., Aricò B. (2005). *Neisseria meningitidis* NadA is a new invasin which promotes bacterial adhesion to and penetration into human epithelial cells. Mol. Microbiol..

[15] Comanducci M., Bambini S., Brunelli B., Adu-Bobie J., Aricò B., Capecchi B., Giuliani M.M., Masignani V., Santini L., Savino S. (2002). NadA, a novel vaccine candidate of *Neisseria meningitidis*. J. Exp. Med..

[16] Findlow J., Borrow R., Snape M.D., Dawson T., Holland A., John T.M., Evans A., Telford K.L., Ypma E., Toneatto D. (2010). Multicenter, open-label, randomized phase II controlled trial of an investigational recombinant meningococcal serogroup B vaccine with and without outer membrane vesicles, administered in infancy. Clin. Infect. Dis..

[17] Bambini S., de Chiara M., Muzzi A., Mora M., Lucidarme J., Brehony C., Borrow R., Masignani V., Comanducci M., Maiden M.C.J. (2014). *Neisseria* adhesin a variation and revised nomenclature scheme. Clin. Vaccine Immunol. CVI.

[18] Dauter Z. (2015). Solving coiled-coil protein structures. IUCrJ.

[19] Dupeux F., Rower M., Seroul G., Blot D., Marquez J.A. (2011). A thermal stability assay can help to estimate the crystallization likelihood of biological samples. Acta Crystallogr. D.

[20] Malito E., Biancucci M., Faleri A., Ferlenghi I., Scarselli M., Maruggi G., Lo Surdo P., Veggi D., Liguori A., Santini L. (2014). Structure of the meningococcal vaccine antigen NadA and epitope mapping of a bactericidal antibody. Proc. Natl. Acad. Sci. USA.

[21] Dale G.E., Oefner C., D’Arcy A. (2003). The protein as a variable in protein crystallization. J. Struct. Biol..

[22] Keenan R.J., Siehl D.L., Gorton R., Castle L.A. (2005). DNA shuffling as a tool for protein crystallization. Proc. Natl. Acad. Sci. USA.

[23] Hartmann M.D., Ridderbusch O., Zeth K., Albrecht R., Testa O., Woolfson D.N., Sauer G., Dunin-Horkawicz S., Lupas A.N., Alvarez B.H. (2009). A coiled-coil motif that sequesters ions to the hydrophobic core. Proc. Natl. Acad. Sci. USA.

[24] Tavano R., Capecchi B., Montanari P., Franzoso S., Marin O., Sztukowska M., Cecchini P., Segat D., Scarselli M., Aricò B. (2011). Mapping of the *Neisseria meningitidis* NadA cell-binding site: Relevance of predicted α-helices in the NH_2_-terminal and dimeric coiled-coil regions. J. Bacteriol..

[25] Berntsson R.P., Smits S.H., Schmitt L., Slotboom D.J., Poolman B. (2010). A structural classification of substrate-binding proteins. FEBS Lett..

[26] Bagnoli F., Fontana M.R., Soldaini E., Mishra R.P., Fiaschi L., Cartocci E., Nardi-Dei V., Ruggiero P., Nosari S., de Falco M.G. (2015). Vaccine composition formulated with a novel TLR7-dependent adjuvant induces high and broad protection against *Staphylococcus aureus*. Proc. Natl. Acad. Sci. USA.

[27] Mishra R.P., Mariotti P., Fiaschi L., Nosari S., Maccari S., Liberatori S., Fontana M.R., Pezzicoli A., de Falco M.G., Falugi F. (2012). Staphylococcus aureus FhuD2 is involved in the early phase of staphylococcal dissemination and generates protective immunity in mice. J. Infect. Dis..

[28] Mariotti P., Malito E., Biancucci M., Lo Surdo P., Mishra R.P., Nardi-Dei V., Savino S., Nissum M., Spraggon G., Grandi G. (2013). Structural and functional characterization of the *Staphylococcus aureus* virulence factor and vaccine candidate FhuD2. Biochem. J..

[29] Podkowa K.J., Briere L.A., Heinrichs D.E., Shilton B.H. (2014). Crystal and solution structure analysis of FhuD2 from *Staphylococcus aureus* in multiple unliganded conformations and bound to ferrioxamine-B. Biochemistry.

[30] Scully I.L., Liberator P.A., Jansen K.U., Anderson A.S. (2014). Covering all the bases: Preclinical development of an effective *Staphylococcus aureus* vaccine. Front. Immunol..

[31] Abate F., Malito E., Cozzi R., Lo Surdo P., Maione D., Bottomley M.J. (2014). Apo, Zn^2+^-bound and Mn^2+^-bound structures reveal ligand-binding properties of SitA from the pathogen *Staphylococcus pseudintermedius*. Biosci. Rep..

[32] Gribenko A., Mosyak L., Ghosh S., Parris K., Svenson K., Moran J., Chu L., Li S., Liu T., Woods V.L. (2013). Three-dimensional structure and biophysical characterization of *Staphylococcus aureus* cell surface antigen-manganese transporter MntC. J. Mol. Biol..

[33] Anderson A.S., Scully I.L., Timofeyeva Y., Murphy E., McNeil L.K., Mininni T., Nunez L., Carriere M., Singer C., Dilts D.A. (2012). *Staphylococcus aureus* manganese transport protein C is a highly conserved cell surface protein that elicits protective immunity against *S. aureus* and *Staphylococcus epidermidis*. J. Infect. Dis..

[34] Ahuja S., Rouge L., Swem D.L., Sudhamsu J., Wu P., Russell S.J., Alexander M.K., Tam C., Nishiyama M., Starovasnik M.A. (2015). Structural analysis of bacterial ABC transporter inhibition by an antibody fragment. Structure.

[35] Plotkin S.A. (2010). Correlates of protection induced by vaccination. Clin. Vaccine Immunol..

[36] Georgiou G., Ippolito G.C., Beausang J., Busse C.E., Wardemann H., Quake S.R. (2014). The promise and challenge of high-throughput sequencing of the antibody repertoire. Nat. Biotechnol..

[37] Huang J., Doria-Rose N.A., Longo N.S., Laub L., Lin C.L., Turk E., Kang B.H., Migueles S.A., Bailer R.T., Mascola J.R. (2013). Isolation of human monoclonal antibodies from peripheral blood B cells. Nat. Protoc..

[38] Wilson P.C., Andrews S.F. (2012). Tools to therapeutically harness the human antibody response. Nat. Rev. Immunol..

[39] Donnarumma D., Bottomley M.J., Malito E., Settembre E., Ferlenghi I., Cozzi R., Bagnoli F., Rappuoli R. (2015). Advanced Vaccine Research.

[40] Malito E., Faleri A., Lo Surdo P., Veggi D., Maruggi G., Grassi E., Cartocci E., Bertoldi I., Genovese A., Santini L. (2013). Defining a protective epitope on factor H binding protein, a key meningococcal virulence factor and vaccine antigen. Proc. Natl. Acad. Sci. USA.

[41] Schneider M.C., Prosser B.E., Caesar J.J., Kugelberg E., Li S., Zhang Q., Quoraishi S., Lovett J.E., Deane J.E., Sim R.B. (2009). *Neisseria meningitidis* recruits factor H using protein mimicry of host carbohydrates. Nature.

[42] Giuntini S., Reason D.C., Granoff D.M. (2011). Complement-mediated bactericidal activity of anti-factor H binding protein monoclonal antibodies against the meningococcus relies upon blocking factor H binding. Infect. Immun..

[43] Chowdary T.K., Cairns T.M., Atanasiu D., Cohen G.H., Eisenberg R.J., Heldwein E.E. (2010). Crystal structure of the conserved herpesvirus fusion regulator complex gH–gL. Nat. Struct. Mol. Biol..

[44] Ciferri C., Chandramouli S., Donnarumma D., Nikitin P.A., Cianfrocco M.A., Gerrein R., Feire A.L., Barnett S.W., Lilja A.E., Rappuoli R. (2015). Structural and biochemical studies of HCMV gH/gL/gO and pentamer reveal mutually exclusive cell entry complexes. Proc. Natl. Acad. Sci. USA.

[45] Kringelum J.V., Nielsen M., Padkjaer S.B., Lund O. (2013). Structural analysis of B-cell epitopes in antibody: Protein complexes. Mol. Immunol..

[46] Griffin L., Lawson A. (2011). Antibody fragments as tools in crystallography. Clin. Exp. Immunol..

[47] Madico G., Welsch J.A., Lewis L.A., McNaughton A., Perlman D.H., Costello C.E., Ngampasutadol J., Vogel U., Granoff D.M., Ram S. (2006). The meningococcal vaccine candidate GNA1870 binds the complement regulatory protein factor H and enhances serum resistance. J. Immunol..

[48] Schneider M.C., Exley R.M., Chan H., Feavers I., Kang Y.H., Sim R.B., Tang C.M. (2006). Functional significance of factor H binding to *Neisseria meningitidis*. J. Immunol..

[49] Jolley K.A., Maiden M.C. (2010). BIGSdb: Scalable analysis of bacterial genome variation at the population level. BMC Bioinform..

[50] Giuliani M.M., Biolchi A., Serruto D., Ferlicca F., Vienken K., Oster P., Rappuoli R., Pizza M., Donnelly J. (2010). Measuring antigen-specific bactericidal responses to a multicomponent vaccine against serogroup B meningococcus. Vaccine.

[51] Masignani V., Comanducci M., Giuliani M.M., Bambini S., Adu-Bobie J., Arico B., Brunelli B., Pieri A., Santini L., Savino S. (2003). Vaccination against *Neisseria meningitidis* using three variants of the lipoprotein GNA1870. J. Exp. Med..

[52] Seib K.L., Serruto D., Oriente F., Delany I., Adu-Bobie J., Veggi D., Arico B., Rappuoli R., Pizza M. (2009). Factor H-binding protein is important for meningococcal survival in human whole blood and serum and in the presence of the antimicrobial peptide LL-37. Infect. Immun..

[53] Cantini F., Veggi D., Dragonetti S., Savino S., Scarselli M., Romagnoli G., Pizza M., Banci L., Rappuoli R. (2009). Solution structure of the factor H-binding protein, a survival factor and protective antigen of *Neisseria meningitidis*. J. Biol. Chem..

[54] Cendron L., Veggi D., Girardi E., Zanotti G. (2011). Structure of the uncomplexed *Neisseria meningitidis* factor H-binding protein fHbp (rLP2086). Acta Crystallogr. Sect. F Struct. Biol. Cryst. Commun..

[55] Mascioni A., Bentley B.E., Camarda R., Dilts D.A., Fink P., Gusarova V., Hoiseth S.K., Jacob J., Lin S.L., Malakian K. (2009). Structural basis for the immunogenic properties of the meningococcal vaccine candidate LP2086. J. Biol. Chem..

[56] Johnson S., Tan L., van der Veen S., Caesar J., Goicoechea De Jorge E., Harding R.J., Bai X., Exley R.M., Ward P.N., Ruivo N. (2012). Design and evaluation of meningococcal vaccines through structure-based modification of host and pathogen molecules. PLoS Pathog..

[57] Scarselli M., Arico B., Brunelli B., Savino S., di Marcello F., Palumbo E., Veggi D., Ciucchi L., Cartocci E., Bottomley M.J. (2011). Rational design of a meningococcal antigen inducing broad protective immunity. Sci. Transl. Med..

[58] Cozzi R., Scarselli M., Ferlenghi I. (2013). Structural vaccinology: A three-dimensional view for vaccine development. Curr. Top. Med. Chem..

[59] Dormitzer P.R., Grandi G., Rappuoli R. (2012). Structural vaccinology starts to deliver. Nat. Rev. Microbiol..

[60] Beernink P.T., Shaughnessy J., Ram S., Granoff D.M. (2010). Impaired immunogenicity of a meningococcal factor H-binding protein vaccine engineered to eliminate factor h binding. Clin. Vaccine Immunol..

[61] Beernink P.T., Shaughnessy J., Braga E.M., Liu Q., Rice P.A., Ram S., Granoff D.M. (2011). A meningococcal factor H binding protein mutant that eliminates factor H binding enhances protective antibody responses to vaccination. J. Immunol..

[62] Beernink P.T., Shaughnessy J., Pajon R., Braga E.M., Ram S., Granoff D.M. (2012). The effect of human factor H on immunogenicity of meningococcal native outer membrane vesicle vaccines with over-expressed factor H binding protein. PLoS Pathog..

[63] Van der Veen S., Johnson S., Jongerius I., Malik T., Genovese A., Santini L., Staunton D., Ufret-Vincenty R.L., Pickering M., Lea S.M. (2014). Non-functional variant 3 factor H binding proteins as meningococcal vaccine candidates. Infect. Immun..

[64] Calmettes C., Alcantara J., Yu R.H., Schryvers A.B., Moraes T.F. (2012). The structural basis of transferrin sequestration by transferrin-binding protein B. Nat. Struct. Mol. Biol..

[65] Noinaj N., Buchanan S.K., Cornelissen C.N. (2012). The transferrin-iron import system from pathogenic *Neisseria* species. Mol. Microbiol..

[66] Noinaj N., Easley N.C., Oke M., Mizuno N., Gumbart J., Boura E., Steere A.N., Zak O., Aisen P., Tajkhorshid E. (2012). Structural basis for iron piracy by pathogenic *Neisseria*. Nature.

[67] Frandoloso R., Martinez-Martinez S., Calmettes C., Fegan J., Costa E., Curran D., Yu R.H., Gutierrez-Martin C.B., Rodriguez-Ferri E.F., Moraes T.F. (2015). Nonbinding site-directed mutants of transferrin binding protein B exhibit enhanced immunogenicity and protective capabilities. Infect. Immun..

[68] Nair H., Nokes D.J., Gessner B.D., Dherani M., Madhi S.A., Singleton R.J., O’Brien K.L., Roca A., Wright P.F., Bruce N. (2010). Global burden of acute lower respiratory infections due to respiratory syncytial virus in young children: A systematic review and meta-analysis. Lancet.

[69] Lozano R., Naghavi M., Foreman K., Lim S., Shibuya K., Aboyans V., Abraham J., Adair T., Aggarwal R., Ahn S.Y. (2012). Global and regional mortality from 235 causes of death for 20 age groups in 1990 and 2010: A systematic analysis for the Global Burden of Disease Study 2010. Lancet.

[70] Falsey A.R., Hennessey P.A., Formica M.A., Cox C., Walsh E.E. (2005). Respiratory syncytial virus infection in elderly and high-risk adults. N. Engl. J. Med..

[71] The IMpact-RSV-Study-Group (1998). Palivizumab, a humanized respiratory syncytial virus monoclonal antibody, reduces hospitalization from respiratory syncytial virus infection in high-risk infants. Pediatrics.

[72] Guvenel A.K., Chiu C., Openshaw P.J. (2014). Current concepts and progress in RSV vaccine development. Expert Rev. Vaccines.

[73] McCarthy M., Villafana T., Stillman E., Esser M.T. (2014). Respiratory syncytial virus protein structure, function and implications for subunit vaccine development. Future Virol..

[74] Anderson R., Huang Y., Langley J.M. (2010). Prospects for defined epitope vaccines for respiratory syncytial virus. Future Microbiol..

[75] McLellan J.S., Ray W.C., Peeples M.E. (2013). Structure and function of respiratory syncytial virus surface glycoproteins. Curr. Top. Microbiol. Immunol..

[76] Calder L.J., Gonzalez-Reyes L., Garcia-Barreno B., Wharton S.A., Skehel J.J., Wiley D.C., Melero J.A. (2000). Electron microscopy of the human respiratory syncytial virus fusion protein and complexes that it forms with monoclonal antibodies. Virology.

[77] Liljeroos L., Krzyzaniak M.A., Helenius A., Butcher S.J. (2013). Architecture of respiratory syncytial virus revealed by electron cryotomography. Proc. Natl. Acad. Sci. USA.

[78] Begona Ruiz-Arguello M., Gonzalez-Reyes L., Calder L.J., Palomo C., Martin D., Saiz M.J., Garcia-Barreno B., Skehel J.J., Melero J.A. (2002). Effect of proteolytic processing at two distinct sites on shape and aggregation of an anchorless fusion protein of human respiratory syncytial virus and fate of the intervening segment. Virology.

[79] Swanson K.A., Settembre E.C., Shaw C.A., Dey A.K., Rappuoli R., Mandl C.W., Dormitzer P.R., Carfi A. (2011). Structural basis for immunization with postfusion respiratory syncytial virus fusion F glycoprotein (RSV F) to elicit high neutralizing antibody titers. Proc. Natl. Acad. Sci. USA.

[80] Wu H., Pfarr D.S., Johnson S., Brewah Y.A., Woods R.M., Patel N.K., White W.I., Young J.F., Kiener P.A. (2007). Development of motavizumab, an ultra-potent antibody for the prevention of respiratory syncytial virus infection in the upper and lower respiratory tract. J. Mol. Biol..

[81] McLellan J.S., Chen M., Chang J.S., Yang Y., Kim A., Graham B.S., Kwong P.D. (2010). Structure of a major antigenic site on the respiratory syncytial virus fusion glycoprotein in complex with neutralizing antibody 101F. J. Virol..

[82] McLellan J.S., Chen M., Kim A., Yang Y., Graham B.S., Kwong P.D. (2010). Structural basis of respiratory syncytial virus neutralization by motavizumab. Nat. Struct. Mol. Biol..

[83] McLellan J.S., Yang Y., Graham B.S., Kwong P.D. (2011). Structure of respiratory syncytial virus fusion glycoprotein in the postfusion conformation reveals preservation of neutralizing epitopes. J. Virol..

[84] Magro M., Mas V., Chappell K., Vazquez M., Cano O., Luque D., Terron M.C., Melero J.A., Palomo C. (2012). Neutralizing antibodies against the preactive form of respiratory syncytial virus fusion protein offer unique possibilities for clinical intervention. Proc. Natl. Acad. Sci. USA.

[85] McLellan J.S., Chen M., Leung S., Graepel K.W., Du X., Yang Y., Zhou T., Baxa U., Yasuda E., Beaumont T. (2013). Structure of RSV fusion glycoprotein trimer bound to a prefusion-specific neutralizing antibody. Science.

[86] McLellan J.S., Chen M., Joyce M.G., Sastry M., Stewart-Jones G.B., Yang Y., Zhang B., Chen L., Srivatsan S., Zheng A. (2013). Structure-based design of a fusion glycoprotein vaccine for respiratory syncytial virus. Science.

[87] McLellan J.S., Correia B.E., Chen M., Yang Y., Graham B.S., Schief W.R., Kwong P.D. (2011). Design and characterization of epitope-scaffold immunogens that present the motavizumab epitope from respiratory syncytial virus. J. Mol. Biol..

[88] Correia B.E., Bates J.T., Loomis R.J., Baneyx G., Carrico C., Jardine J.G., Rupert P., Correnti C., Kalyuzhniy O., Vittal V. (2014). Proof of principle for epitope-focused vaccine design. Nature.

[89] Koff W.C., Russell N.D., Walport M., Feinberg M.B., Shiver J.W., Karim S.A., Walker B.D., McGlynn M.G., Nweneka C.V., Nabel G.J. (2013). Accelerating the development of a safe and effective HIV vaccine: HIV vaccine case study for the Decade of Vaccines. Vaccine.

[90] Ward A.B., Wilson I.A. (2015). Insights into the trimeric HIV-1 envelope glycoprotein structure. Trends Biochem. Sci..

[91] Rerks-Ngarm S., Pitisuttithum P., Nitayaphan S., Kaewkungwal J., Chiu J., Paris R., Premsri N., Namwat C., de Souza M., Adams E. (2009). Vaccination with ALVAC and AIDSVAX to prevent HIV-1 infection in Thailand. N. Engl. J. Med..

[92] Haynes B.F., Gilbert P.B., McElrath M.J., Zolla-Pazner S., Tomaras G.D., Alam S.M., Evans D.T., Montefiori D.C., Karnasuta C., Sutthent R. (2012). Immune-correlates analysis of an HIV-1 vaccine efficacy trial. N. Engl. J. Med..

[93] West A.P., Scharf L., Scheid J.F., Klein F., Bjorkman P.J., Nussenzweig M.C. (2014). Structural insights on the role of antibodies in HIV-1 vaccine and therapy. Cell.

[94] Kwong P.D., Mascola J.R., Nabel G.J. (2013). Broadly neutralizing antibodies and the search for an HIV-1 vaccine: The end of the beginning. Nat. Rev. Immunol..

[95] Kwong P.D., Wyatt R., Robinson J., Sweet R.W., Sodroski J., Hendrickson W.A. (1998). Structure of an HIV gp120 envelope glycoprotein in complex with the CD4 receptor and a neutralizing human antibody. Nature.

[96] Correia B.E., Ban Y.E., Holmes M.A., Xu H., Ellingson K., Kraft Z., Carrico C., Boni E., Sather D.N., Zenobia C. (2010). Computational design of epitope-scaffolds allows induction of antibodies specific for a poorly immunogenic HIV vaccine epitope. Structure.

[97] Azoitei M.L., Correia B.E., Ban Y.-E.A., Carrico C., Kalyuzhniy O., Chen L., Schroeter A., Huang P.-S., Mclellan J.S., Kwong P.D. (2011). Computation-guided backbone grafting of a discontinuous motif onto a protein scaffold. Science.

[98] Ofek G., Guenaga F.J., Schief W.R., Skinner J., Baker D., Wyatt R., Kwong P.D. (2010). Elicitation of structure-specific antibodies by epitope scaffolds. Proc. Natl. Acad. Sci. USA.

[99] Dey B., Svehla K., Xu L., Wycuff D., Zhou T., Voss G., Phogat A., Chakrabarti B.K., Li Y., Shaw G. (2009). Structure-based stabilization of HIV-1 gp120 enhances humoral immune responses to the induced co-receptor binding site. PLoS Pathog..

[100] Kassa A., Dey A.K., Sarkar P., Labranche C., Go E.P., Clark D.F., Sun Y., Nandi A., Hartog K., Desaire H. (2013). Stabilizing exposure of conserved epitopes by structure guided insertion of disulfide bond in HIV-1 envelope glycoprotein. PLoS ONE.

[101] Jardine J., Julien J.P., Menis S., Ota T., Kalyuzhniy O., McGuire A., Sok D., Huang P.S., MacPherson S., Jones M. (2013). Rational HIV immunogen design to target specific germline B cell receptors. Science.

[102] Julien J.P., Cupo A., Sok D., Stanfield R.L., Lyumkis D., Deller M.C., Klasse P.J., Burton D.R., Sanders R.W., Moore J.P. (2013). Crystal structure of a soluble cleaved HIV-1 envelope trimer. Science.

[103] Pancera M., Zhou T., Druz A., Georgiev I.S., Soto C., Gorman J., Huang J., Acharya P., Chuang G.Y., Ofek G. (2014). Structure and immune recognition of trimeric pre-fusion HIV-1 Env. Nature.

[104] Lyumkis D., Julien J.P., de Val N., Cupo A., Potter C.S., Klasse P.J., Burton D.R., Sanders R.W., Moore J.P., Carragher B. (2013). Cryo-EM structure of a fully glycosylated soluble cleaved HIV-1 envelope trimer. Science.

[105] Sanders R.W., Derking R., Cupo A., Julien J.P., Yasmeen A., de Val N., Kim H.J., Blattner C., de la Pena A.T., Korzun J. (2013). A next-generation cleaved, soluble HIV-1 Env trimer, BG505 SOSIP.664 gp140, expresses multiple epitopes for broadly neutralizing but not non-neutralizing antibodies. PLoS Pathog..

[106] Hol W., Verlinde C. (2006). Macromolecular Crystallography and Medicine.

[107] Tickle I., Sharff A., Vinkovic M., Yon J., Jhoti H. (2004). High-throughput protein crystallography and drug discovery. Chem. Soc. Rev..

[108] Berman H.M., Kleywegt G.J., Nakamura H., Markley J.L. (2012). The protein data bank at 40: Reflecting on the past to prepare for the future. Structure (Lond., Engl.).

[109] Terwilliger T.C., Stuart D., Yokoyama S. (2009). Lessons from structural genomics. Ann. Rev. Biophys..

[110] Bernal J.D., Crowfoot D. (1934). X-ray photographs of crystalline pepsin. Nature.

[111] Kendrew J.C., Bodo G., Dintzis H.M., Parrish R.G., Wyckoff H., Phillips D.C. (1958). A three-dimensional model of the myoglobin molecule obtained by X-ray analysis. Nature.

[112] Kendrew J.C., Dickerson R.E., Strandberg B.E., Hart R.G., Davies D.R., Phillips D.C., Shore V.C. (1960). Structure of myoglobin: A three-dimensional Fourier synthesis at 2 Å. resolution. Nature.

[113] Perutz M.F., Rossmann M.G., Cullis A.F., Muirhead H., Will G., North A.C. (1960). Structure of haemoglobin: A three-dimensional Fourier synthesis at 5.5-A. resolution, obtained by X-ray analysis. Nature.

[114] Blake C.C., Fenn R.H., North A.C., Phillips D.C., Poljak R.J. (1962). Structure of lysozyme. A Fourier map of the electron density at 6 angstrom resolution obtained by X-ray diffraction. Nature.

[115] Blake C.C., Koenig D.F., Mair G.A., North A.C., Phillips D.C., Sarma V.R. (1965). Structure of hen egg-white lysozyme. A three-dimensional Fourier synthesis at 2 Angstrom resolution. Nature.

[116] Garman E.F. (2014). Developments in X-ray crystallographic structure determination of biological macromolecules. Science.

[117] Chayen N.E., Saridakis E. (2008). Protein crystallization: From purified protein to diffraction-quality crystal. Nat. Methods.

[118] Bukowska M.A., Grütter M.G. (2013). New concepts and aids to facilitate crystallization. Curr. Opin. Struct. Biol..

[119] McPherson A., Cudney B. (2014). Optimization of crystallization conditions for biological macromolecules. Acta Crystallogr. Sect. F Struct. Biol. Commun..

[120] Chandonia J.-M., Brenner S.E. (2006). The impact of structural genomics: Expectations and outcomes. Science.

[121] Hendrickson W.A. (2007). Impact of structures from the protein structure initiative. Structure (Lond., Engl.).

[122] Rupp B. (2003). High-throughput crystallography at an affordable cost: The TB structural genomics consortium crystallization facility. Acc. Chem. Res..

[123] Vedadi M., Arrowsmith C.H., Allali-Hassani A., Senisterra G., Wasney G.A. (2010). Biophysical characterization of recombinant proteins: A key to higher structural genomics success. J. Struct. Biol..

[124] Yumerefendi H., Tarendeau F., Mas P.J., Hart D.J. (2010). ESPRIT: An automated, library-based method for mapping and soluble expression of protein domains from challenging targets. J. Struct. Biol..

[125] Reich S., Puckey L.H., Cheetham C.L., Harris R., Ali A.A., Bhattacharyya U., Maclagan K., Powell K.A., Prodromou C., Pearl L.H. (2006). Combinatorial domain hunting: An effective approach for the identification of soluble protein domains adaptable to high-throughput applications. Protein Sci..

[126] Derewenda Z.S. (2010). Application of protein engineering to enhance crystallizability and improve crystal properties. Acta Crystallogr. D.

[127] Lamazares E., Clemente I., Bueno M., Velazquez-Campoy A., Sancho J. (2015). Rational stabilization of complex proteins: A divide and combine approach. Sci. Rep..

[128] Derewenda Z.S., Vekilov P.G. (2006). Entropy and surface engineering in protein crystallization. Acta Crystallogr. D.

[129] Zou Y., Weis W.I., Kobilka B.K. (2012). N-terminal T4 lysozyme fusion facilitates crystallization of a G protein coupled receptor. PLoS ONE.

[130] Chun E., Thompson A.A., Liu W., Roth C.B., Griffith M.T., Katritch V., Kunken J., Xu F., Cherezov V., Hanson M.A. (2012). Fusion partner toolchest for the stabilization and crystallization of G protein-coupled receptors. Structure.

[131] Hassell A.M., An G., Bledsoe R.K., Bynum J.M., Carter H.L., Deng S.-J.J., Gampe R.T., Grisard T.E., Madauss K.P., Nolte R.T. (2007). Crystallization of protein-ligand complexes. Acta Crystallogr. D.

[132] Tereshko V., Uysal S., Koide A., Margalef K., Koide S., Kossiakoff A.A. (2008). Toward chaperone-assisted crystallography: Protein engineering enhancement of crystal packing and X-ray phasing capabilities of a camelid single-domain antibody (VHH) scaffold. Protein Sci. Publ. Protein Soc..

[133] Wernimont A., Edwards A. (2009). *In situ* proteolysis to generate crystals for structure determination: An update. PLoS ONE.

[134] D’Arcy A., Villard F., Marsh M. (2007). An automated microseed matrix-screening method for protein crystallization. Acta crystallogr. Sect. D Biol. Crystallogr..

[135] Bergfors T. (2003). Seeds to crystals. J. Struct. Biol..

[136] Walter T.S., Meier C., Assenberg R., Au K.-F., Ren J., Verma A., Nettleship J.E., Owens R.J., Stuart D.I., Grimes J.M. (2006). Lysine methylation as a routine rescue strategy for protein crystallization. Structure (Lond., Engl.).

[137] Kim Y., Quartey P., Li H., Volkart L., Hatzos C., Chang C., Nocek B., Cuff M., Osipiuk J., Tan K. (2008). Large-scale evaluation of protein reductive methylation for improving protein crystallization. Nat. Methods.

[138] Newman J., Bolton E.E., Mueller-Dieckmann J., Fazio V.J., Gallagher D.T., Lovell D., Luft J.R., Peat T.S., Ratcliffe D., Sayle R.A. (2012). On the need for an international effort to capture, share and use crystallization screening data. Acta Crystallogr. F.

[139] Fazio V.J., Peat T.S., Newman J. (2014). A drunken search in crystallization space. Acta Crystallogr. F.

[140] Fusco D., Barnum T.J., Bruno A.E., Luft J.R., Snell E.H., Mukherjee S., Charbonneau P. (2014). Statistical analysis of crystallization database links protein physico-chemical features with crystallization mechanisms. PLoS ONE.

[141] Ng J.T., Dekker C., Kroemer M., Osborne M., von Delft F. (2014). Using textons to rank crystallization droplets by the likely presence of crystals. Acta Crystallogr. D.

[142] Dauter Z., Jaskolski M., Wlodawer A. (2010). Impact of synchrotron radiation on macromolecular crystallography: A personal view. J. Synchrotron Radiat..

[143] Broennimann C., Eikenberry E.F., Henrich B., Horisberger R., Huelsen G., Pohl E., Schmitt B., Schulze-Briese C., Suzuki M., Tomizaki T. (2006). The PILATUS 1M detector. J. Synchrotron Radiat..

[144] Mueller M., Wang M., Schulze-Briese C. (2012). Optimal fine φ-slicing for single-photon-counting pixel detectors. Acta Crystallogr. D.

[145] Gabadinho J., Beteva A., Guijarro M., Rey-Bakaikoa V., Spruce D., Bowler M.W., Brockhauser S., Flot D., Gordon E.J., Hall D.R. (2010). MxCuBE: A synchrotron beamline control environment customized for macromolecular crystallography experiments. J. Synchrotron Radiat..

[146] Helliwell J.R., Mitchell E.P. (2015). Synchrotron radiation macromolecular crystallography: Science and spin-offs. IUCrJ.

[147] Malbet-Monaco S., Leonard G.A., Mitchell E.P., Gordon E.J. (2013). How the ESRF helps industry and how they help the ESRF. Acta Crystallogr. D.

[148] Schlichting I. (2015). Serial femtosecond crystallography: The first five years. IUCrJ.

[149] Boutet S., Lomb L., Williams G.J., Barends T.R., Aquila A., Doak R.B., Weierstall U., DePonte D.P., Steinbrener J., Shoeman R.L. (2012). High-resolution protein structure determination by serial femtosecond crystallography. Science.

[150] Liu W., Wacker D., Gati C., Han G.W., James D., Wang D., Nelson G., Weierstall U., Katritch V., Barty A. (2013). Serial femtosecond crystallography of G protein-coupled receptors. Science.

[151] Tenboer J., Basu S., Zatsepin N., Pande K., Milathianaki D., Frank M., Hunter M., Boutet S., Williams G.J., Koglin J.E. (2014). Time-resolved serial crystallography captures high-resolution intermediates of photoactive yellow protein. Science.

[152] Hendrickson W.A. (1991). Determination of macromolecular structures from anomalous diffraction of synchrotron radiation. Science.

[153] Garman E., Murray J.W. (2003). Heavy-atom derivatization. Acta Crystallogr. D.

[154] Rould M.A. (2007). The same but different: Isomorphous methods for phasing and high-throughput ligand screening. Methods Mol. Biol..

[155] Gonzalez A., von Delft F., Liddington R.C., Bakolitsa C. (2005). Two-wavelength MAD phasing and radiation damage: A case study. J. Synchrotron Radiat..

[156] Dodson E. (2003). Is it jolly SAD?. Acta Crystallogr. Sect. D Biol. Crystallogr..

[157] Dauter Z. (2002). One-and-a-half wavelength approach. Acta Crystallogr. D.

[158] Abendroth J., Gardberg A.S., Robinson J.I., Christensen J.S., Staker B.L., Myler P.J., Stewart L.J., Edwards T.E. (2011). SAD phasing using iodide ions in a high-throughput structural genomics environment. J. Struct. Funct. Genomics.

[159] Dauter Z. (2002). New approaches to high-throughput phasing. Curr. Opin. Struct. Biol..

[160] Dauter Z., Li M., Wlodawer A. (2001). Practical experience with the use of halides for phasing macromolecular structures: A powerful tool for structural genomics. Acta Crystallogr. D.

[161] Nagem R.A., Dauter Z., Polikarpov I. (2001). Protein crystal structure solution by fast incorporation of negatively and positively charged anomalous scatterers. Acta Crystallogr. D.

[162] Scapin G. (2013). Molecular replacement then and now. Acta Crystallogr. D.

[163] Evans P., McCoy A. (2008). An introduction to molecular replacement. Acta Crystallogr. D.

[164] Abergel C. (2013). Molecular replacement: Tricks and treats. Acta Crystallogr. D.

[165] McCoy A.J., Grosse-Kunstleve R.W., Adams P.D., Winn M.D., Storoni L.C., Read R.J. (2007). Phaser crystallographic software. J. Appl. Crystallogr..

[166] Vagin A., Teplyakov A. (1997). MOLREP: An automated program for molecular replacement. J. Appl. Crystallogr..

[167] Long F., Vagin A.A., Young P., Murshudov G.N. (2008). BALBES: A molecular-replacement pipeline. Acta Crystallogr. Sect. D Biol. Crystallogr..

[168] Keegan R.M., Winn M.D. (2008). MrBUMP: An automated pipeline for molecular replacement. Acta Crystallogr. Sect. D Biol. Crystallogr..

[169] Stokes-Rees I., Sliz P. (2010). Protein structure determination by exhaustive search of Protein Data Bank derived databases. Proc. Natl. Acad. Sci. USA.

[170] Afonine P.V., Grosse-Kunstleve R.W., Echols N., Headd J.J., Moriarty N.W., Mustyakimov M., Terwilliger T.C., Urzhumtsev A., Zwart P.H., Adams P.D. (2012). Towards automated crystallographic structure refinement with phenix.refine. Acta Crystallogr. D.

[171] Winn M.D., Ballard C.C., Cowtan K.D., Dodson E.J., Emsley P., Evans P.R., Keegan R.M., Krissinel E.B., Leslie A.G., McCoy A. (2011). Overview of the CCP4 suite and current developments. Acta Crystallogr. Sect. D Biol. Crystallogr..

[172] Morin A., Eisenbraun B., Key J., Sanschagrin P.C., Timony M.A., Ottaviano M., Sliz P. (2013). Collaboration gets the most out of software. eLife.

[173] Aoki S.T., Settembre E.C., Trask S.D., Greenberg H.B., Harrison S.C., Dormitzer P.R. (2009). Structure of rotavirus outer-layer protein VP7 bound with a neutralizing Fab. Science.

[174] Li X., Mooney P., Zheng S., Booth C.R., Braunfeld M.B., Gubbens S., Agard D.A., Cheng Y. (2013). Electron counting and beam-induced motion correction enable near-atomic-resolution single-particle cryo-EM. Nat. Methods.

[175] Zhang X., Settembre E., Xu C., Dormitzer P.R., Bellamy R., Harrison S.C., Grigorieff N. (2008). Near-atomic resolution using electron cryomicroscopy and single-particle reconstruction. Proc. Natl. Acad. Sci. USA.

[176] Brown A., Amunts A., Bai X.C., Sugimoto Y., Edwards P.C., Murshudov G., Scheres S.H., Ramakrishnan V. (2014). Structure of the large ribosomal subunit from human mitochondria. Science.

[177] Liao M., Cao E., Julius D., Cheng Y. (2013). Structure of the TRPV1 ion channel determined by electron cryo-microscopy. Nature.

[178] Wu S., Avila-Sakar A., Kim J., Booth D.S., Greenberg C.H., Rossi A., Liao M., Li X., Alian A., Griner S.L. (2012). Fabs enable single particle cryoEM studies of small proteins. Structure.

[179] Zhang Q., Noble K.A., Mao Y., Young N.L., Sathe S.K., Roux K.H., Marshall A.G. (2013). Rapid screening for potential epitopes reactive with a polycolonal antibody by solution-phase H/D exchange monitored by FT-ICR mass spectrometry. J. Am. Soc. Mass Spectrom..

[180] Landgraf R.R., Chalmers M.J., Griffin P.R. (2012). Automated hydrogen/deuterium exchange electron transfer dissociation high resolution mass spectrometry measured at single-amide resolution. J. Am. Soc. Mass Spectrom..

